# Tetraboron MR framework with *ortho*-B-*π*-B pattern enables high-efficiency yellow narrowband OLEDs

**DOI:** 10.1093/nsr/nwag389

**Published:** 2026-06-24

**Authors:** Shuqi Zhang, Yongliu Yang, Zhangli Cheng, Tongyuan Zhang, Jie Li, Moyuan Li, Jia Yu, Xiaochun Fan, Kai Wang, Xiaohong Zhang

**Affiliations:** Institute of Functional Nano & Soft Materials (FUNSOM), Soochow University, Suzhou 215123, China; Institute of Functional Nano & Soft Materials (FUNSOM), Soochow University, Suzhou 215123, China; Institute of Functional Nano & Soft Materials (FUNSOM), Soochow University, Suzhou 215123, China; Institute of Functional Nano & Soft Materials (FUNSOM), Soochow University, Suzhou 215123, China; School of Materials Science and Chemical Engineering, Ningbo University, Ningbo 315211, China; Institute of Functional Nano & Soft Materials (FUNSOM), Soochow University, Suzhou 215123, China; Institute of Functional Nano & Soft Materials (FUNSOM), Soochow University, Suzhou 215123, China; Institute of Functional Nano & Soft Materials (FUNSOM), Soochow University, Suzhou 215123, China; Jiangsu Key Laboratory of Advanced Negative Carbon Technologies, Soochow University, Suzhou 215123, China; Institute of Functional Nano & Soft Materials (FUNSOM), Soochow University, Suzhou 215123, China; State Key Laboratory of Bioinspired Interfacial Materials Science, Soochow University, Suzhou 215123, China; Institute of Functional Nano & Soft Materials (FUNSOM), Soochow University, Suzhou 215123, China; Jiangsu Key Laboratory of Advanced Negative Carbon Technologies, Soochow University, Suzhou 215123, China

**Keywords:** multiple resonance, narrowband emission, one-shot quadruple borylation, *ortho*-B-*π*-B pattern

## Abstract

Multiple resonance (MR) frameworks with *ortho*-boron-*π*-boron (B-*π*-B) patterns are promising alternatives to those with *para*-B-*π*-B ones for realizing bathochromic narrowband emission, but such cases have been rarely reported, and suffer from significant synthetic challenges. Here, a novel tetraboron MR framework ICZ4B incorporating an *ortho*-B-*π*-B pattern was constructed by using indolo[2,3-c]carbazole as the central building block. The well-defined borylation active sites facilitate an efficient one-shot quadruple borylation. ICZ4B not only exhibits bright yellow emission with a small full width at half maximum of 24 nm, but also achieves an improved reverse intersystem crossing rate of 4.7 × 10^4^ s^−1^. The non-sensitized device realizes an outstanding external quantum efficiency of 36.5%, representing one of the highest results at long wavelengths. This study not only presents a feasible pathway for realizing MR frameworks with *ortho*-B-*π*-B patterns via delicate selection of key precursors, but also offers insights for optimizing synthetic strategies for complex MR frameworks.

## Teaser

A tetraboron MR framework featuring ortho B-π-B pattern was synthesized from indolo[2,3-c]carbazole via one-shot borylation, exhibiting yellow emission at 562 nm and a record external quantum efficiency of 36.5% in non-sensitized organic light-emitting diode s.

## INTRODUCTION

As organic light-emitting diode (OLED) technology continues to evolve, the demand for ultra-high-definition displays is gradually increasing in recent years [1,2]. This trend has imposed more stringent requirements on emitting materials, particularly concerning their color purity, which is directly governed by emission bandwidths. Among the available OLED material library, organoboron emitters with distinct multiple resonance (MR) effect are widely regarded as the most promising candidates due to their inherent narrowband characteristic. In such a classical emitter, e.g. DABNA-1, the electron-withdrawing boron (B) atoms are purposely embedded in polyaromatics adjacent to electron-donating nitrogen (N) atoms to induce atomic-level orbital character, thereby enabling minimized vibronic coupling and structural relaxation and allowing narrowband emission [3]. Moreover, the thermally activated delayed fluorescence (TADF) characteristic generally emerges as well due to their small singlet-triplet energy differences (Δ*E*_ST_), permitting efficient triplet harvesting. However, the mutually inhibited electron delocalization of such *ortho*-positioned B/N patterns results in relatively large optical bandgaps. Thus, the simple MR frameworks almost emit within a narrow range at short wavelengths (e.g. blue to sky blue). Although fusing multiple such frameworks following *meta*-B-*π*-B patterned extension could substantially enhance TADF properties and reduce bandwidths, the emission position barely shifts [4].

In 2021, Yasuda *et al.* introduced an epoch-making MR framework BBCz-R, displaying pure-red emission with an extremely small full width at half maximum (FWHM) of 21 nm [5]. This breakthrough results from the strategic rearrangement of the heteroatomic pattern, wherein two *para*-positioned B atoms within the skeleton (i.e. *para*-B-*π*-B pattern) can significantly enhance the electron-withdrawing strength. Crucially, the resultant hybridized *π*-/non-bonding orbital character maintains suppressed vibronic coupling between the ground and excited states, thereby preserving the narrowband characteristic [6]. To date, the *para*-B-*π*-B pattern has continually facilitated the implementation of highly emissive narrowband MR emitters at the long-wavelength region [7–9]. Nevertheless, they almost face a common shortcoming, i.e. poor TADF properties. This is because the *para*-B-*π*-B distributions cannot effectively break the *π*-conjugation, thus leading to a considerably large energy difference between the lowest unoccupied molecular orbital (LUMO) and LUMO + 1 levels, which hinders Δ*E*_ST_ reduction in MR systems [10]. In addition, the synthesis of *para*-B-*π*-B-based frameworks relies heavily on the organolithium-mediated borylation, which not only suffers from poor reaction yields and complicated purification procedure, but also limits the molecular design room [5].

Recently, Hatakeyama *et al.* reported the exceptional obtainment of a ‘core-shell’ type MR emitter, Δ-DABNA-TB, with three adjacent B atoms as the core, which presents promising red narrowband emission [11]. These results indicate that the *ortho-*B-*π*-B pattern could be an alternative paradigm of the *para*-B-*π*-B pattern due to their equal resonance effect. Nevertheless, mechanism of the borylation procedure for Δ-DABNA-TB remains unclear, possibly involving several unpredictable retro-bora-Friedel−Craft processes, hindering its further derivations. Most recently, Zheng *et al.* reported the second case of an MR emitter integrating a *para*-B-*π*-B pattern; however, the synthesis is complicated, requires the assistance of an organolithium reagent, and results in poor yields [12]. Given the exceedingly limited reports on *ortho-*B-*π*-B-based MR emitters, the development of systematic molecular frameworks, together with the optimization of the associated synthetic methodologies, are urgently needed at this stage, albeit accompanied by considerable challenges. Additionally, another issue is that both the reported examples cannot deliver decent device performance in absence of sensitizers because of their poor TADF characteristics.

**Figure 1.**
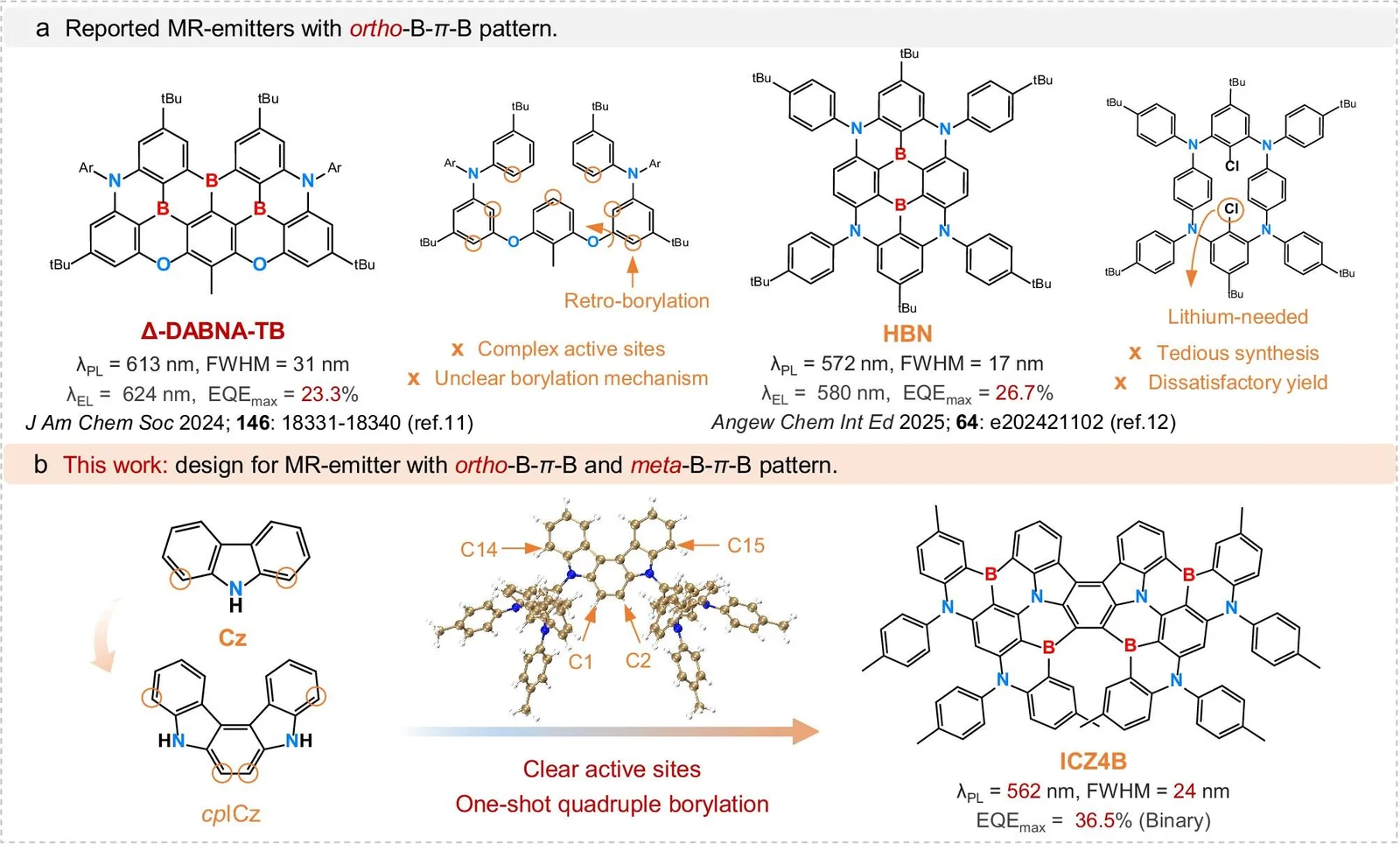
(a) Previous works of MR-emitters with *ortho*-B-*π*-B pattern and (b) design concept of this work: MR-emitter with *ortho*-B-*π*-B and *meta*-B-*π*-B pattern.

In this work, we exploit indolo[2,3-c]carbazole (*cp*ICz) as the central building block to realize a facile and rational synthesis of an MR emitter with *ortho*-B-*π*-B pattern. The strong electron-donating character of the *cp*ICz unit natively provides abundant active sites for borylation, including two crucial sites adjacently located on the central benzene ring. By rationally cooperating with peripherally orientated diphenylamine units, the proof-of-concept molecule ICZ4B was successfully synthesized through an efficient one-shot quadruple borylation. As shown in Fig. 1, ICZ4B comprises four B atoms and six N atoms, which are all strong driving forces for attenuating the bonding orbital character, and enhancing the narrowband characteristic. More importantly, the two B atoms in the cove region follow the *ortho* B-*π*-B pattern, which are together expected to be a stronger electron-deficient center than a conventional sole B atom and reduce the optical bandgap; on the other hand, the other two at the bilateral regions independently form conventional *meta*-B-*π*-B patterns, which could help to break the *π*-conjugation and reduce Δ*E*_ST_. As a result, ICZ4B exhibits bright yellow emission at 562 nm in dilute toluene solution, with a small FWHM of 24 nm (energy unit: 93 meV) and evident TADF properties. A non-sensitized OLED device using ICZ4B emitter displays an exceptional maximum external quantum efficiency (EQE_max_) of up to 36.5% with electroluminescence (EL) peak at 568 nm. This work offers important insights into the structural assembly design for the design and the facile synthesis of challenging MR frameworks involving the unique *ortho*-B-*π*-B pattern, thus expanding the chemical space for the development of organoboron MR family.

## RESULTS AND DISCUSSION

### Synthesis and characterization

**Figure 2.**
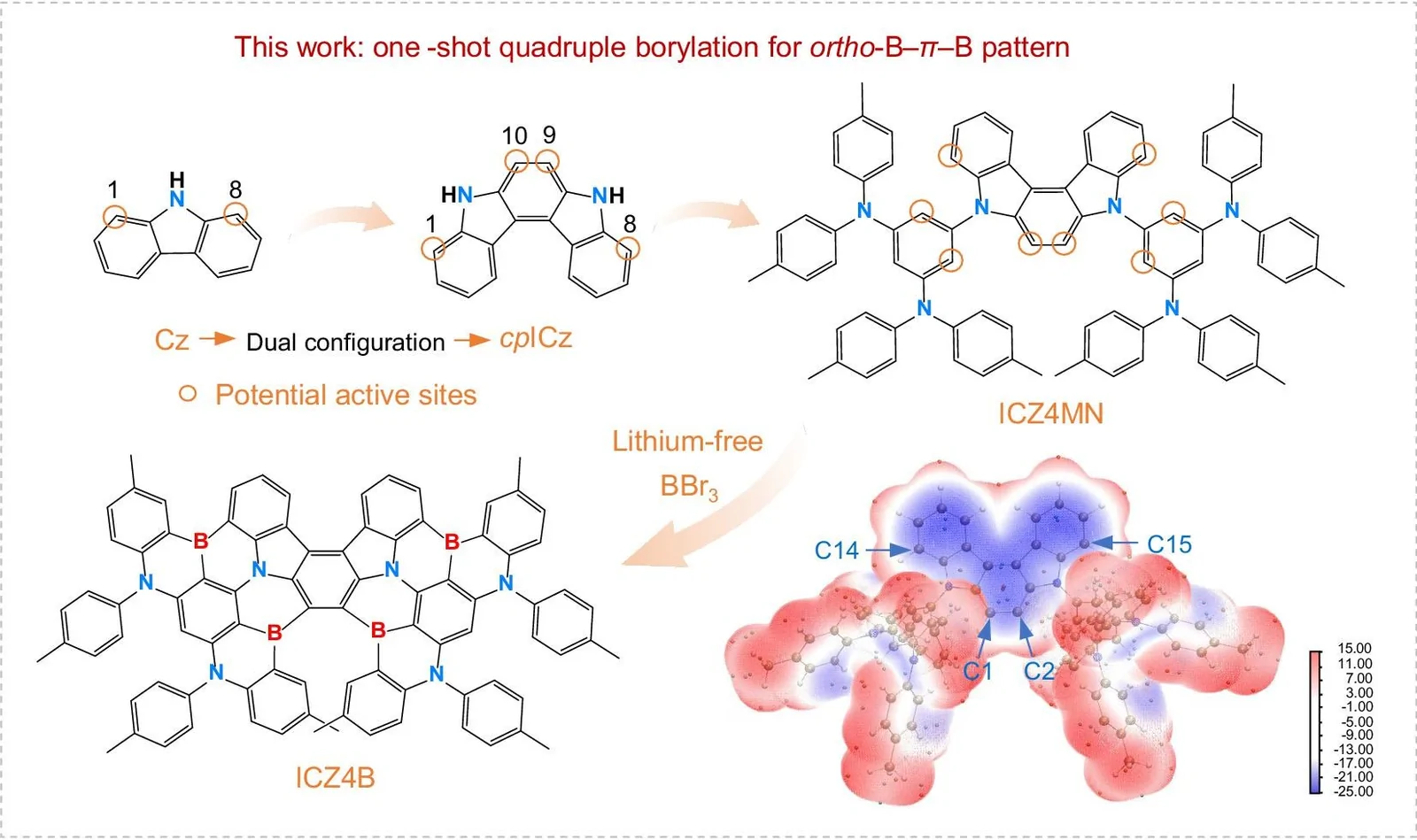
Design and synthetic strategy for target molecule (orange circles indicate potential active sites), along with the electrostatic potential distribution of the intermediate ICZ4MN.

The molecular assembly strategy and detailed synthetic route for the target molecule ICZ4B are illustrated in Fig. 2 and Fig. S2. Previous studies have demonstrated that in a single carbazole unit, the C1 and C8 positions are the most favorable sites to support electrophilic borylation reaction [13]. Inspired by these, a strategically-fused indolocarbazole isomer *cp*ICz, which inherits all the *ortho*-active positions of its monomeric carbazole, including two adjacent sites (i.e. C9 and C10) for borylation, is designed as a basic scaffold for the construction of an *ortho* B-*π*-B pattern. Notably, although *cp*ICz has previously served as a building block for MR frameworks, it only offered a single borylation site [14–17]. Here, to achieve site-selective borylation at all these positions, diphenylamine moieties are additionally introduced as directing groups to assist the insertion of boron halides. The key borylated-precursor ICZ4MN was obtained in high yields via two steps of Buchwald-Hartwig cross-coupling reactions. As revealed by the electrostatic potential (ESP) distribution of ICZ4MN, the adjacent C1/C2 and separated C14/C15 sites possess nearly identical and minimal ESP values, indicating that they are the most probable sites for borylation [18,19]. Finally, a one-shot quadruple borylation was achieved by using 40 equivalents of boron tribromide (BBr_3_) in a solution of *o*-dichlorobenzene at 210°C, affording the target molecule ICZ4B in a satisfactory yield with high selectivity. It is noteworthy that this structural design enables facile one-shot construction of a large multiple-boron-embedded framework consisting of two adjacent boron atoms in the central ring, without requiring high-pressure reactors or more reactive boron sources such as boron triiodide (BI_3_). Detailed structural characterization data of all new compounds are provided in the Supporting Information. Thermogravimetric analysis result indicates that ICZ4B exhibits excellent thermal stability, with a decomposition temperature as high as 599°C, confirming its suitability for vacuum evaporation processes (Fig. S3). In particular, a single-crystal sample of the target compound was prepared using a liquid-phase diffusion method with a mixed solvent system of tetrahydrofuran and methanol, and analyzed *via* single-crystal X-ray diffraction to confirm its chemical structure (Table S1). As shown in Fig. S4, a fully-fused polycyclic framework with four B atoms precisely inserted is characterized as predicted, confirming the obtainment of ICZ4B. Owing to the intense steric hindrance caused by adjacent boron-embedded moieties, the whole skeleton of ICZ4B is highly distorted with a large dihedral angle of 46.85° in the cove region. The pronounced distortion and the presence of non-fused peripheral methylbenzene rings, can efficiently suppress the intermolecular *π*–*π* stacking of ICZ4B, as evidenced by the limited stacking-overlap. Therefore, the aggregation-caused quenching typically observed for MR molecules is expected to be mitigated for ICZ4B, despite its significantly extended skeleton [20,21].

### Theoretical calculations

**Figure 3.**
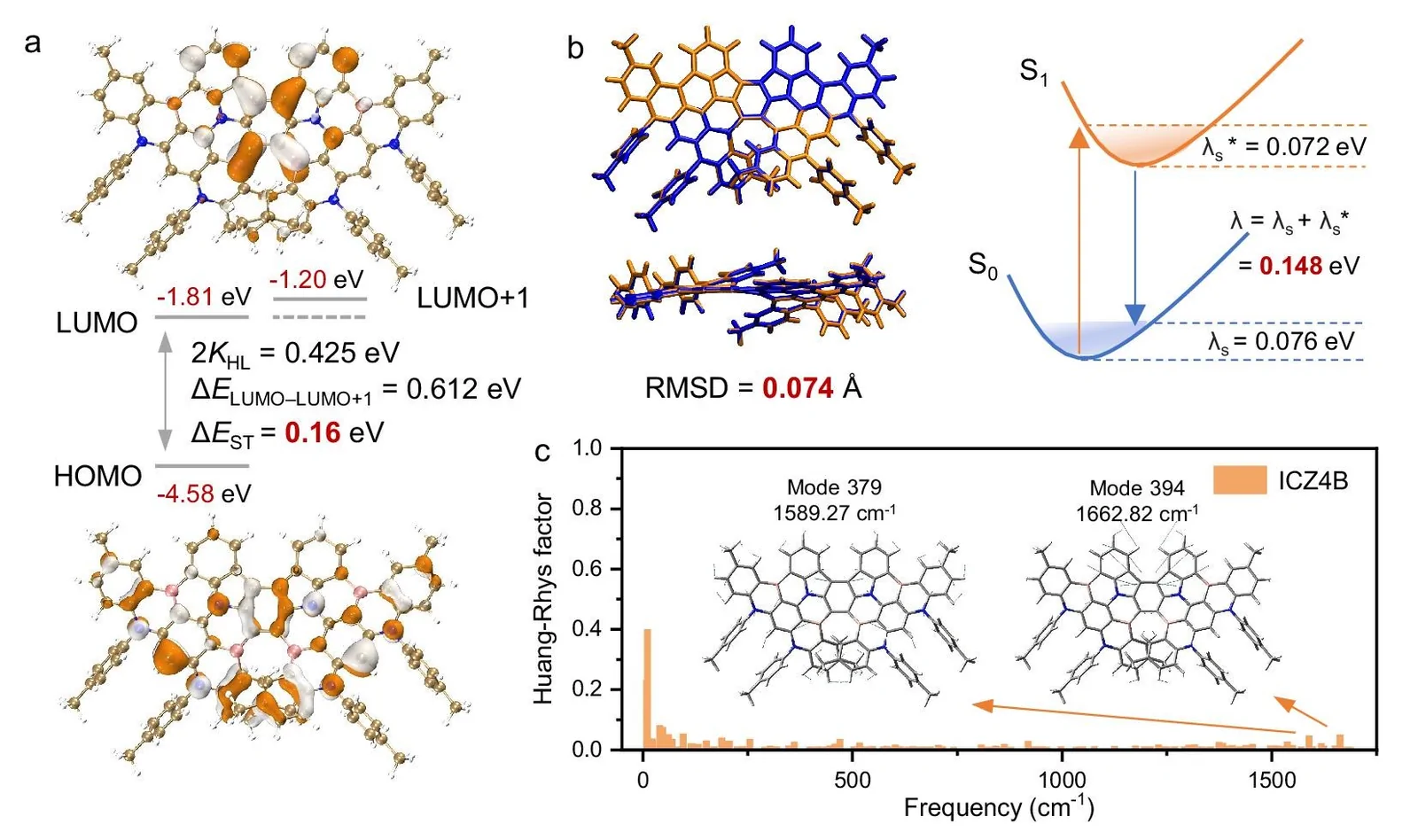
(a) The FMO distributions and estimated Δ*E*_ST_ based on the combined consideration of the influence of 2*K*_HL_ and Δ*E*_LUMO−LUMO+1_. (b) RMSD and reorganization energy and (c) calculated HR factor for the S_1_ → S_0_ transition (inset: stretching vibration modes).

To investigate the electronic properties of ICZ4B, density functional theory (DFT) calculations were performed at the PEB0/6-31G(d) level. The molecular frontier orbital (FMO) distribution and corresponding energy levels were firstly calculated. As shown in Fig. 3a, the overall impression is that the highest occupied molecular orbital (HOMO) of ICZ4B is delocalized over nearly the entire framework, whereas the LUMO is predominantly localized on the *cp*ICz region, leading to a small exchange energy (2*K*_HL_) of 0.425 eV. The differentiated HOMO/LUMO distributions can be attributed to the specific arrangement of heteroatoms, in which all four B atoms are integrated onto *cp*ICz, thereby significantly enhancing the electron-withdrawing strength of this moiety. In terms of detailed orbital characteristics, the core region of the framework is dominated by both *ortho*-B-*π*-B and *para*-N-*π*-N patterns, which impart a bonding character manifested as continuous electron density along the C–C and C–B bonds. In contrast, the shell region of the framework is composed of the conventional *ortho*-B-*π*-N pattern, resulting in electron density alternately localized on atoms, indicative of a non-bonding character. The hybrid bonding/non-bonding character has been recognized for suppressing the vibronic coupling and structural relaxation, thereby enabling the narrowband feature inherent to ICZ4B [6]. More importantly, it also contributes to a reduced energy gap of 0.61 eV between the LUMO and LUMO + 1 (Δ*E*_LUMO−LUMO+1_), eventually giving rise to a small enough Δ*E*_ST_ of 0.16 eV for ICZ4B, as predicted by the novel physical picture proposed in our previous literature [10].

Investigations were further conducted on the excited-state electronic properties of ICZ4B using the time-dependent DFT (TD-DFT) method (Fig. S8). The difference-density plot of the S_1_ state exhibits an FMO-dominated distribution, where the hole and electron wavefunctions correspond to the HOMO and LUMO, respectively, suggesting an obvious nonbonding character. Notably, besides T_1_ state, there are another two triplet excited states (i.e. T_2_ and T_3_) lying below/close to the S_1_ state, with spin-orbit coupling values higher than that between S_1_ and T_1_, which would help to build multiple reverse intersystem crossing (RISC) channels for efficient triplet exciton harvest. The formation of the dense intermediate triplet states is attributed to the extended tetraboron framework and the integrated hybrid heteroatomic patterns, which induce distinguishing transition components within ICZ4B.

To further obtain in-depth insight into the structural reorganization of ICZ4B upon excitation, the geometric displacement and detailed vibrational relaxation in the ground (S_0_) and S_1_ states were examined. As shown in Fig. 3b, the optimized geometry of the S_1_ state exhibits substantial overlap with that of its S_0_ state, resulting in an extremely small root mean square displacement value of 0.074 Å and reorganization energy (λ) of 0.148 eV. This suggests that the deviation between the lowest lying energy points of the S_1_ and S_0_ potential energy surfaces is minimized, which is beneficial for achieving a narrow spectral profile. Moreover, analysis of the Huang-Rhys (HR) factors for the S_1_ → S_0_ transition reveals that, except for two dominant vibrational modes in the low-frequency range, the HR factors for all other vibrational modes across the spectrum are less than 0.1, suggesting limited vibronic coupling between the S_1_ and S_0_ states. The two principal vibrational modes in the low-frequency range, with HR factors of 0.22 and 0.39, are observed at 9.12 and 11.13 cm^−1^ (Fig. S9), which correspond to the twisting vibrations of the methylbenzene ring located at the periphery of the ICZ4B framework, respectively. Given that these segments scarcely participate in orbital delocalization and display very low vibrational frequencies, they are unlikely to induce significant spectral broadening. The suppressed vibronic coupling and structural relaxation can be ascribed both to the rigid framework and the hybrid heteroatomic patterns, ultimately endowing ICZ4B with an expectedly narrowband emission. To support our claim, we further analyzed two control molecules, SN-PAH and CzDB, featuring related structures to ICZ4B (see their chemical structures detailed in Fig. S10). Estimation results indicate that FMOs of SN-PAH and CzDB are clearly dominated by bonding and non-bonding characters, respectively. As shown in Fig. S12, the summed HR factor of ICZ4B is slightly higher than that of CzDB but remains substantially lower than that of SN-PAH, especially in the high-frequency region, indicating only a slight enhancement in vibronic coupling resulting from the hybrid bonding/non-bonding character. These results further demonstrate that the hybrid heteroatomic pattern remains advantageous for preserving the narrowband emission feature of ICZ4B.

### Photophysical properties

**Figure 4.**
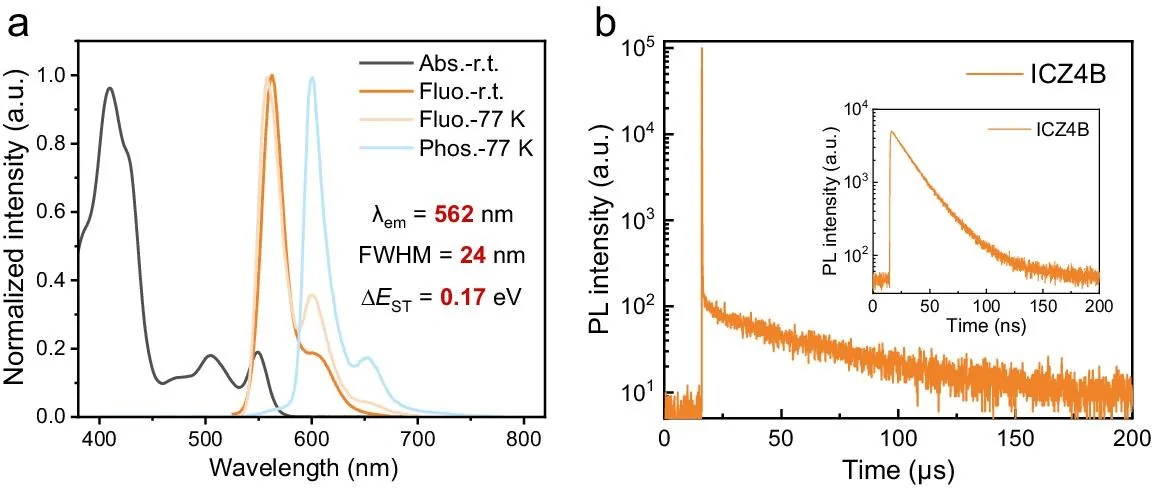
(a) Ultraviolet‒visible absorption, fluorescence spectra at room temperature and fluorescence and phosphorescence spectra at 77 K in dilute toluene and (b) transient PL decay spectra in the 1 wt%-doped film.

The photophysical properties of ICZ4B were firstly investigated in dilute toluene at 10^−5^ M. As shown in Fig. 4a, ICZ4B exhibits intense absorption peaks around 400 nm, which are ascribed to the localized transition from fused segments, while the lowest absorption band was observed at 550 nm with moderate intensity. Corresponding to this absorption band, a sharp bright yellow emission at 562 nm was achieved in its fluorescence spectrum. The Stokes shift was estimated to be as small as 12 nm, indicating minimized structural relaxation upon excitation, which is consistent with theoretical predictions. Moreover, the FWHM of 24 nm (energy unit: 93 meV) represents one of the narrowest emission bands within the similar region. The slight shoulder appearing around 600 nm could be assigned to the contribution of high-frequency vibration modes, i.e. in-plane stretching vibrations of the whole skeleton and out-plane scissoring vibrations dominated by the *cp*ICz segment (Fig. 3c). Furthermore, according to low-temperature fluorescent and phosphorescent spectra, the S_1_ and T_1_ energy levels of ICZ4B were calculated to be 2.29 and 2.12 eV, resulting in a moderate ∆*E*_ST_ of 0.17 eV, aligning well with our estimations. Although this value is mediocre compared with those short-wavelength MR emitters, such as *ν*-DABNA series, it remains competitive among those of the reported long-wavelength MR emitters with emission peaks beyond 560 nm (Table S4), enough for emerging obvious TADF features.

To further evaluate the TADF property of ICZ4B, transient photoluminescence (PL) decay was performed in 1 wt% doped film using 2-(9,9′-spirobi[fluoren]-3-yl)-4,6-diphenyl-1,3,5-triazine (SF3-TRZ) as the host material. The PL quantum yield (PLQY) of this doped film was estimated at 99%, indicating a remarkably high exciton utilization efficiency under photoexcitation. Based on the decay curves in Fig. 4b, the prompt and delayed lifetimes were fitted to be 20.9 ns and 30.7 μs, respectively. The detailed kinetic parameters were further calculated to reveal the exciton utilization. As listed in Table 1 and Table S3, ICZ4B exhibits a large radiative decay rate (*k*_r_) of 3.2 × 10^7^ s^−1^, which is significantly higher than the non-radiative decay rate (*k*_nr_) of 3.3 × 10^5^ s^−1^, thereby rendering the fluorescence process the predominant channel for exciton decay. Moreover, a moderate RISC rate (*k*_RISC_) of 4.7 × 10^4^ s^−1^ was observed for ICZ4B [22]. To our knowledge, this rate exceeds that of most reported long-wavelength MR emitters with emission peaks beyond 560 nm, as these emitters typically possess extensive *π*-conjugated frameworks, which impede the achievement of efficient TADF properties (Table S4). To gain insight into the enhanced RISC process, temperature-dependent transient PL measurements of 1 wt% doped film were performed from 180 to 300 K (Fig. S15). The activation energy was determined to be 76.7 meV, smaller than the ∆*E*_ST_ of 170 meV obtained from low-temperature spectroscopy, indicating that the T_1_-S_1_ channel is not the dominant one in the overall RISC process. Instead, intermediate triplet excited states closer to the S_1_ state are likely involved, which contribute to exciton upconversion and thereby enabling multiple RISC pathways. Furthermore, benefiting from the extended *π*-skeleton, ICZ4B also exhibits an exceptional horizontal molecular orientation ratio (Θ*_//_*) of up to 92%, which can further enhance the photon extraction efficiency, thereby improving overall efficiency of the corresponding OLED devices.

**Table 1.** Photophysical parameters and kinetic constant data of ICZ4B.

	λ_abs_^a^ (nm)	λ_em_^a^ (nm)	∆*E*_ST_^b^ (eV)	FWHM^a^ (nm)/(eV)	*Φ*_PL_^c^ (%)	*τ*_p_^d^ (ns)	*τ*_d_^e^ (μs)	*k*_RISC_^f^ (10^4^ s^−1^)	*k*_r_^g^ (10^7^ s^−1^)	Θ*_/__/_*^h^ (%)
ICZ4B	550	562	0.17	24/0.093	99	20.9	30.7	4.7	3.2	92

a Measured in toluene at 300 K

b *∆E*_ST_ = *E*_S1_–*E*_T1_

c PLQY determined from 1 wt%-doped films in SF3-TRZ at 300 K under a N_2_ atmosphere

d Lifetime of prompt fluorescence component

e Lifetime of delayed fluorescence component

f The rate constant of reverse intersystem crossing (T_1_ → S_1_)

g The rate constant of fluorescence radiative decay (S_1_ → S_0_)

h The horizontal molecular orientation ratio.

### Device performance

**Figure 5.**
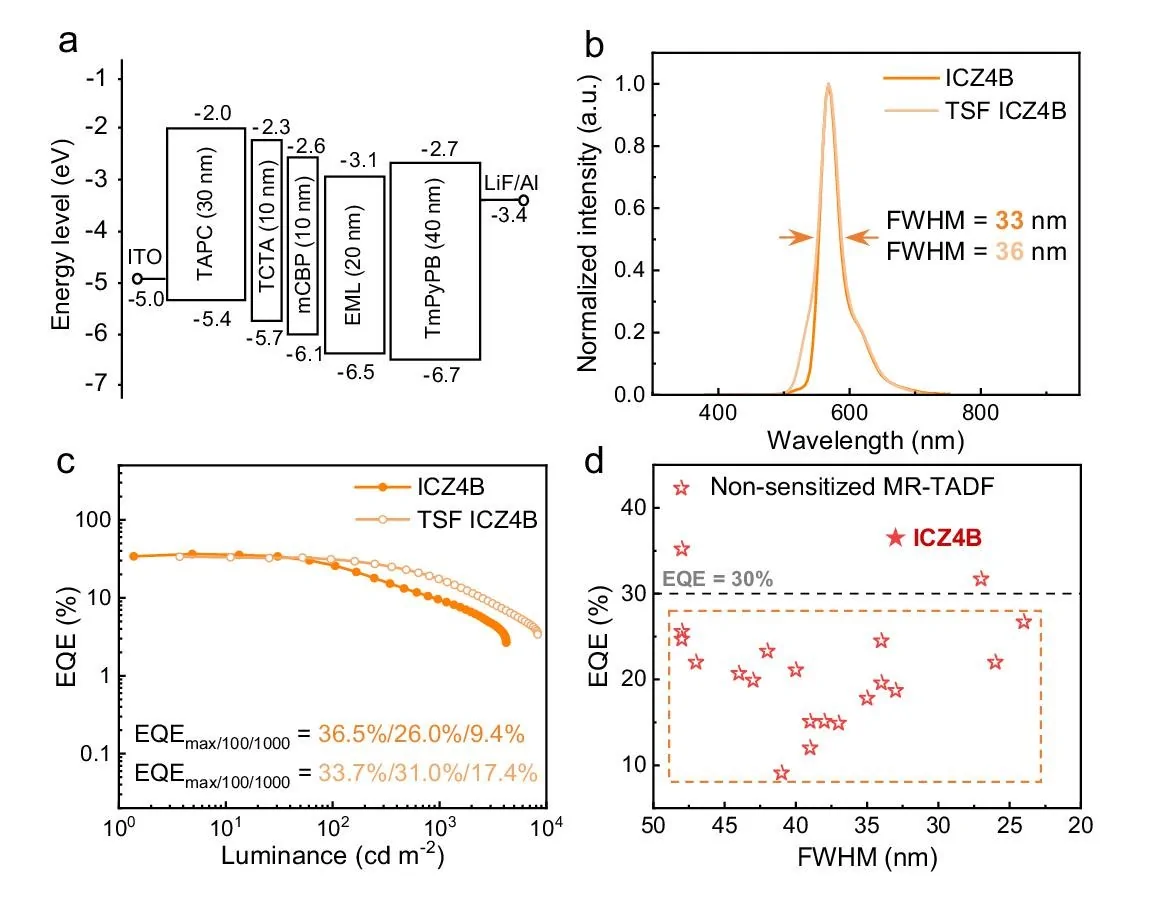
(a) Device structure. (b) EL spectra of binary and ternary devices. (c) EQE-luminance curves of 1 wt%-doped binary and ternary devices and (d) plot of EQE_max_ against FWHM for representative long-wavelength (λ_PL_ > 560 nm) MR-TADF-based OLEDs ever reported.

Inspired by the favorable photophysical properties, the EL performance of ICZ4B-based devices was further evaluated. As shown in Fig. 5a, the optimized device structure is consisting of ITO/4,4′-cyclohexylidenebis[*N,N*-bis(4-methylphenyl)aniline] (TAPC, 30 nm)/(tris(4-carbazolyl-9-ylphenyl)amine (TCTA, 10 nm)/1,3-di(9*H*-carbazol-9-yl)benzene (mCBP, 10 nm)/emitting layer (EML, 20 nm)/3,3′-[5′-[3-(3-pyridinyl)phenyl] [1,1′:3′,1″-terphenyl]-3,3″-diyl]bispyridine (TmPyPB, 40 nm)/LiF (1 nm)/Al. The energy level diagram and molecular structures of involved materials are provided in Figs S17 and S18. Considering the adequate TADF property of ICZ4B, the EML was initially designed with a non-sensitized binary system, utilizing SF3-TRZ as the sole host. As illustrated in Fig. 5 and Fig. S20, 1 wt%-doped device exhibits a sharp EL spectrum at 568 nm with a narrow FWHM of 33 nm (energy unit: 126 meV). Benefitting from the near-unity PLQY and large Θ_//_factor, this device also demonstrates an outstanding EQE_max_ of up to 36.5%, along with corresponding maximum current efficiency (CE_max_) and maximum power efficiency (PE_max_) of 133 cd A^−1^ and 116 lm W^−1^, respectively. As summarized in Fig. 5d and Table S6, this performance is highly competitive among the reported devices based on long-wavelength MR emitters, especially in terms of its record-high EQE value [5–9,11,12,23–30]. Moreover, these binary devices demonstrate low sensitivity to the doping concentration, achieving high EQE_max_ exceeding 33% and maintaining stable FWHMs of 33 nm across all the tested doped devices. This can be attributed to the spatially steric hindrance of the SF3-TRZ host, together with the highly distorted framework of ICZ4B itself, which mitigates the concentration quenching typically observed for MR emitters. Although these devices perform well at low luminance level (e.g. below 100 cd m^−2^), their EQEs drop significantly at elevated luminance levels (e.g. ~1000 cd m^−2^), indicating that the exciton utilization efficiency of ICZ4B remains limited at high exciton density. To elucidate the effect of energy level alignment on charge carrier dynamics in the non-sensitized devices, single-carrier devices based on the host matrix SF3-TRZ with/without 1 wt% ICZ4B were further fabricated. As shown in Fig. S21a, the closely aligned LUMO energy levels of ICZ4B and SF3-TRZ (−3.11 vs. −3.10 eV) allow similar current density–voltage (*J*–*V*) characteristics in both electron-only devices, indicating barely affected electron injection and electron transport properties. On the other hand, their hole-only devices exhibit distinct dynamic behaviors (Fig. S21b). This can be attributed to the substantial HOMO energy gap of 1.24 eV between ICZ4B and SF3-TRZ (−5.30 vs. −6.54 eV), resulting in a pronounced hole trapping effect. Here, although the shallow HOMO energy level of ICZ4B promotes the hole injection from the adjacent layer, and thus allows a slightly enhanced current density at low voltages, the resulting deep HOMO well traps the mobile hole, leading to a much-suppressed hole transport behavior at high voltages. Therefore, at low current density, electrons and holes are relatively balanced, allowing excitons to be effectively confined within ICZ4B and converted efficiently into photons, thereby yielding high EQE at low luminance. While at high current densities, charge transport becomes electron-dominated, and the limited concentration of the emitter leads to pronounced exciton quenching, resulting in a marked efficiency roll-off at high luminance. To alleviate this dilemma, a ternary TADF-assisted-fluorescence (TSF) technology was employed. Here, L-DABNA-1, a yellow-green MR emitter in our previous work, was selected as the TADF assistant due to the rapid *k*_RISC_ rate and pronounced overlap of its PL spectrum with the absorption of ICZ4B [31]. As shown in Fig. 5 and Fig. S23, the EL profile of the ternary device is nearly identical to that of the optimal binary device with ICZ4B and clearly distinct from that of the control binary device only with L-DABNA-1, indicating that the terminal emitter ICZ4B dominates the overall EL characteristics in the ternary TSF device. Although the EQE_max_ of 33.7% for this device is slightly inferior to the best result of the binary devices, the efficiency roll-off was significantly improved. An EQE of 17.4% is recorded at 1000 cd cm^−2^, which is almost double that of the binary device, revealing the effectiveness of the TSF strategy in enhancing device performance at practical brightness.

Furthermore, we evaluate the operational stability of ICZ4B with a ternary EML containing 4 wt% L-DABNA-1 as the sensitizer (see Fig. S24 for details). The device lifetime with the luminance decay to 80% of the initial luminance (1000 cd m^−2^) (LT_80_) reaches up to 114.8 h, slightly higher than that of the control binary device with only 4 wt% L-DABNA-1 as the dopant in the EML (LT_80_ = 104.9 h), demonstrating the significance of the TSF system for improving device operational stability.

## CONCLUSION

In summary, a tetraboron MR framework featuring *ortho*-B-*π*-B pattern was designed by utilizing the *cp*ICZ group with well-defined active sites. The target molecule ICZ4B was synthesized via an organolithium-free one-shot quadruple borylation with satisfactory yield, overcoming the challenge of synthesizing *ortho*-B-*π*-B MR frameworks. Theoretical calculations indicate that four boron atoms localized at the *cp*ICZ core significantly enhance electron-withdrawing properties, while the hybrid bonding/non-bonding character suppresses the vibronic coupling and structural relaxation, together enabling ideal bathochromic narrowband feature of ICZ4B. In toluene solution, ICZ4B displayed a sharp yellow emission at 562 nm with an extremely narrow FWHM of 24 nm (93 meV). Meanwhile, benefitting from the relatively small Δ*E*_ST_ value and multiple RISC channels, the doped film exhibited a fast *k*_RISC_ rate of 4.7 × 10^4^ s^−1^. The non-sensitized device using ICZ4B as emitter achieved a record high EQE_max_ up to 36.5% with a narrow FWHM of 33 nm (126 meV), corresponding to a CE_max_ exceeding 130 cd A^−1^. In addition, the TSF-based ternary device successfully suppresses roll-off to below 50% at 1000 cd cm^−2^. This work not only provides a novel tetraboron MR framework featuring a unique *ortho*-B-*π*-B pattern, but also offers insights for guiding the design of synthetic approaches for MR frameworks with hybrid heteroatomic patterns.

## METHODS

^1^H NMR spectra were recorded on a Bruker 400 spectrometer at room temperature. Mass spectra were recorded on a Bruker Autoflex MALDI-TOF mass spectrometer. Diffraction data were collected on a Rigaku R-AXISRAPID diffractometer in ω-scan mode with graphite-monochromator MoK*α* or CuK*α* radiation. The structure determination was solved with direct methods via the SHELXTL programs and refined with full-matrix least squares on F2. The CIF crystallographic information for ICZ4B has been deposited at the Cambridge Center for Crystallographic Center (CCDC https://www.ccdc.cam.ac.uk/structures/) under deposition numbers 2392596.

DFT and TD-DFT calculations were performed by using Gaussian 09 with the B3LYP/PBE0/6-31G(d) basis set. The calculation of natural transition orbitals were analyzed using a multifunctional wavefunction analyzer (Multiwfn 3.7).

Details of photophysical tests, fabrication/measurement of electroluminescent devices, and cyclic voltammetry measurements are provided in the Supplementary Data.

## Supplementary Material

nwag389_Supplemental_File
