# Supplementary material for: Tetraboron MR framework with ortho-B-π-B pattern enables high-efficiency yellow narrowband OLEDs
Source: Natl Sci Rev. 2026 Jun 24;13(16):nwag389. doi: 10.1093/nsr/nwag389 (PMC13525712; doi:10.1093/nsr/nwag389)
Supplement: Supplementary file 1 [file nwag389_supplemental_file.pdf]

Supplementary data

Tetraboron MR framework with *ortho*-B-π-B pattern enables high-efficiency yellow narrowband OLEDs

ShuQi Zhang[1], YongLiu Yang[1], ZhangLi Cheng[1], TongYuan Zhang[1], Jie Li[2], MoYuan Li[1], Jia Yu[1], XiaoChun Fan[1,4,*], Kai Wang[1,3,*] and XiaoHong Zhang[1,4,*]

[1]Institute of Functional Nano & Soft Materials (FUNSOM), Soochow University, Suzhou215123, China;

[2]School of Materials Science and Chemical Engineering, Ningbo University, Ningbo 315211, China;

[3]State Key Laboratory of Bioinspired Interfacial Materials Science, Soochow University, Suzhou 215123, China and [4]Jiangsu Key Laboratory of Advanced Negative Carbon Technologies, Soochow University, Suzhou 215123, China

***Corresponding authors.** E-mails: xcfan@suda.edu.cn; wkai@suda.edu.cn; xiaohong_zhang@suda.edu.cn.

Table of Contents

1.General Information..S3

2.Synthesis..S7

3. Crystal structure analysis...S9

4. Theoretical calculations...S11

5. Photophysical Properties..S14

6. Electroluminescence Properties...S17

General information

Synthesis. The chemicals and reagents purchased commercially were used without further purification unless otherwise stated. The main synthesis was performed using standard Schlenk techniques under a nitrogen atmosphere, and the target molecule was further purified by sublimation. ^{1}H and ^{13}C NMR spectra were measured on a Bruker 400/101 MHz spectrometer with tetramethylsilane (TMS) as the internal standard. Mass analyses were recorded by an Autoflex MALDI-TOF mass spectrometer.

Photophysical measurements. UV–Vis absorption and photoluminescence (PL) spectra were recorded by using a Hitachi ultraviolet–visible (UV–Vis) spectrophotometer U-3010 and a Hitachi fluorescence spectrometer F-4600, respectively. The PL quantum efficiency (PLQY) was measured via an absolute PL quantum yield measurement system (C9920-02, Hamamatsu Photonics) in glovebox with an excitation wavelength of 300 nm. Transient fluorescence decays were measured with Quantaurus-Tau fluorescence lifetime spectrometer (C11367-32, Hamamatsu Photonics) with an excitation wavelength of 373 nm and a pulse width of 100 ps.

Theoretical calculation. The front molecular orbitals (FMOs) of the optimized ground state and excited state were calculated with time-dependent density function theory (TD-DFT) employing the PEB0/6-31g(d,p) basis set in the Gaussian 16 program package. All these quantum-chemistry calculations were performed by the Gaussian 16 software package and based on the optimized ground-state geometries.

In a two-electron three-orbital active space, five important spin-adapted configurations can be constructed: $^1\Phi_{HH}$, $^3\Phi_{H\to L}$, $^3\Phi_{H\to L+1}$, $^1\Phi_{H\to L}$ and $^1\Phi_{H\to L+1}$,

$$|S_0\rangle = |\,^1\Phi_{\mathrm{HH}}\rangle = \left|\chi_{\mathrm{H}}^{\alpha}\chi_{\mathrm{H}}^{\beta}\right\rangle \tag{S1}$$

$$|S_1\rangle = c_{^1\Phi_{H\to L}}|^1\Phi_{H\to L}\rangle + c_{^1\Phi_{H\to L+1}}|^1\Phi_{H\to L+1}\rangle \tag{S2}$$

$$|^1\Phi_{H\to L}\rangle = N\left\{\left|\chi_{\mathrm{H}}^{\alpha}\chi_{\mathrm{L}}^{\beta}\right\rangle + \left|\chi_{\mathrm{L}}^{\alpha}\chi_{\mathrm{H}}^{\beta}\right\rangle\right\}, |^1\Phi_{H\to L+1}\rangle = N\left\{\left|\chi_{\mathrm{H}}^{\alpha}\chi_{\mathrm{L+1}}^{\beta}\right\rangle + \left|\chi_{\mathrm{L+1}}^{\alpha}\chi_{\mathrm{H}}^{\beta}\right\rangle\right\} \tag{S3}$$

$$|T_1\rangle = c_{^3\Phi_{H\to L}}|^3\Phi_{H\to L}\rangle + c_{^3\Phi_{H\to L+1}}|^3\Phi_{H\to L+1}\rangle \tag{S4}$$

$$|^3\Phi_{H\to L}\rangle = N\left\{\left|\chi_{\mathrm{H}}^{\alpha}\chi_{\mathrm{L}}^{\beta}\right\rangle - \left|\chi_{\mathrm{L}}^{\alpha}\chi_{\mathrm{H}}^{\beta}\right\rangle\right\}, |^3\Phi_{H\to L+1}\rangle = N\left\{\left|\chi_{\mathrm{H}}^{\alpha}\chi_{\mathrm{L+1}}^{\beta}\right\rangle - \left|\chi_{\mathrm{L+1}}^{\alpha}\chi_{\mathrm{H}}^{\beta}\right\rangle\right\} \tag{S5}$$

where the superscripts 1 and 3 denote singlet and triplet spin states, respectively, N is

the normalization factor, α and β denote electrons with different spins, and χ_H, χ_L, and χ_{L+1} indicate the HOMO, LUMO, and LUMO+1 molecular orbitals, respectively. Here, according to the Slater-Condon rule and spin restriction, the electronic interaction (*i.e.*, the off-diagonal element of the effective Hamiltonian) between ${}^1\Phi_{H\to L}$ and ${}^1\Phi_{H\to L+1}$ (or ${}^3\Phi_{H\to L}$ and ${}^3\Phi_{H\to L+1}$) can be expressed as:

$$\langle {}^1\Phi_{H\to L}|\hat{H}|{}^1\Phi_{H\to L+1}\rangle = \langle \chi_H\chi_L|\chi_H\chi_{L+1}\rangle + \langle \chi_H\chi_L|\chi_{L+1}\chi_H\rangle = J_3 + K_3 \qquad \text{(S6)}$$

$$\langle {}^3\Phi_{H\to L}|\hat{H}|{}^3\Phi_{H\to L+1}\rangle = \langle \chi_H\chi_L|\chi_H\chi_{L+1}\rangle - \langle \chi_H\chi_L|\chi_{L+1}\chi_H\rangle = J_3 - K_3 \qquad \text{(S7)}$$

where J_3 and K_3 are both two-electron integrals. Since the two-electron integrals J_3 and K_3 usually have the same sign, their cancellation in Eq. S7 leads to a small $\langle {}^3\Phi_{H\to L}|\hat{H}|{}^3\Phi_{H\to L+1}\rangle$ term. To effectively incorporate the effect of the interactions among all electronic configurations on the S_1 state, the off-diagonal element of the effective Hamiltonian (shown in Figure S1), is set to parameter V.

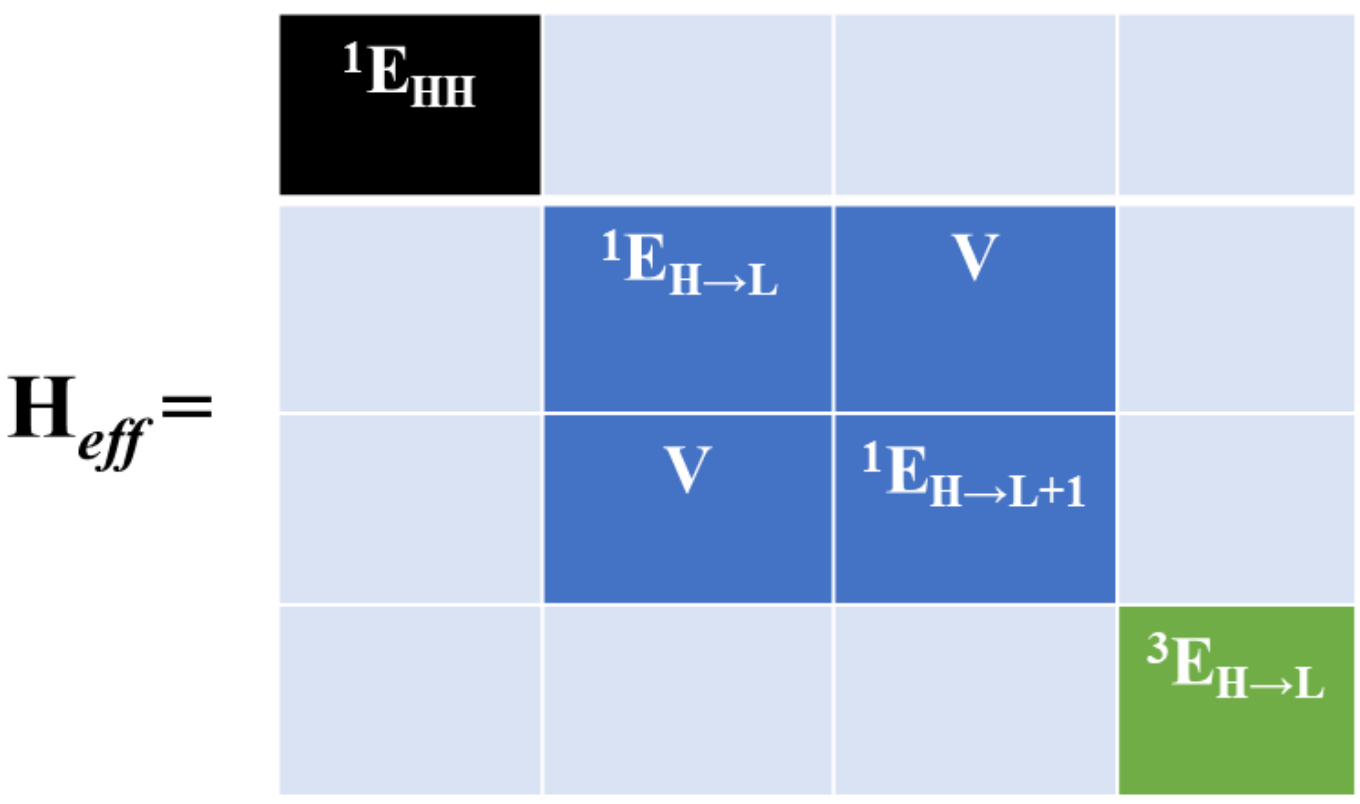

Figure S1 | Illustration of the effective Hamiltonian. ${}^1E_{HH}$, ${}^1E_{H\to L}$, ${}^1E_{H\to L+1}$, and ${}^3E_{H\to L}$ denote the energies for the ${}^1\Phi_{HH}$, ${}^1\Phi_{H\to L}$, ${}^1\Phi_{H\to L+1}$, and ${}^3\Phi_{H\to L}$ configurations, respectively.

The interactions between the configurations can decrease the energy of the S_1 state by ΔE_{shift}. By solving the 2×2 matrix based on ${}^1\Phi_{H\to L}$ and ${}^1\Phi_{H\to L+1}$, we have:

$$E_{S_1} = {}^1E_{H\to L} + \frac{({}^1E_{H\to L+1} - {}^1E_{H\to L}) - \sqrt{({}^1E_{H\to L+1} - {}^1E_{H\to L})^2 + 4(V)^2}}{2} \qquad \text{(S8)}$$

$$\Delta E_{\text{shift}} = \frac{({}^1E_{H\to L+1} - {}^1E_{H\to L}) - \sqrt{({}^1E_{H\to L+1} - {}^1E_{H\to L})^2 + 4(V)^2}}{2} \qquad \text{(S9)}$$

Here,

$$^{1}E_{H\to L} = h_{\mathrm{HH}} + h_{\mathrm{LL}} + J_{\mathrm{HL}} + K_{\mathrm{HL}} \quad \text{(S10)}$$

$$^{1}E_{H\to L+1} = h_{\mathrm{HH}} + h_{\mathrm{L+1L+1}} + J_{\mathrm{HL+1}} + K_{\mathrm{HL+1}} \quad \text{(S11)}$$

where h_{HH}, h_{LL} and $h_{\mathrm{L+1L+1}}$ are the one-electron integrals corresponding to the HOMO, LUMO and LUMO+1, respectively, and K and J are the exchange and Coulomb integrals between two molecular orbitals. The energy difference between the two configurations in Eqs. S11 and S10 can be approximately expressed as the energy gap between LUMO+1 and LUMO:

$$^{1}E_{H\to L+1} - {}^{1}E_{H\to L} \approx E_{\mathrm{LUMO+1}} - E_{\mathrm{LUMO}} \equiv \Delta E_{\mathrm{LUMO-LUMO+1}} \quad \text{(S12)}$$

Hence, $\Delta E_{\mathrm{shift}}$ can be rewritten as:

$$\Delta E_{\mathrm{shift}} \approx \frac{\Delta E_{\mathrm{LUMO-LUMO+1}} - \sqrt{\Delta E_{\mathrm{LUMO-LUMO+1}}{}^{2} + 4V^{2}}}{2} - \frac{2V^{2}}{\Delta E_{\mathrm{LUMO-LUMO+1}} + \sqrt{\Delta E_{\mathrm{LUMO-LUMO+1}}{}^{2} + 4V^{2}}} \quad \text{(S13)}$$

For the T_1 state:

$$E_{\mathrm{T_1}} \approx {}^{3}E_{H\to L} = h_{\mathrm{HH}} + h_{\mathrm{LL}} + J_{\mathrm{HL}} - K_{\mathrm{HL}} \quad \text{(S14)}$$

ΔE_{S1T1} can be rewritten as:

$$\Delta E_{\mathrm{S1T1}} = E_{\mathrm{S_1}} - E_{\mathrm{T_1}} \approx 2K_{HL} - \frac{2V^{2}}{\sqrt{\Delta E_{\mathrm{LUMO-LUMO+1}}{}^{2} + 4V^{2}} + \Delta E_{\mathrm{LUMO-LUMO+1}}} \quad \text{(S15)}$$

According to our previous work, parameter V can be replaced with a function of $2K_{\mathrm{HL}}$:

$$V = f(2K_{\mathrm{HL}}) = (a \times 2\,K_{\mathrm{HL}}) + b \quad \text{(S16)}$$

where the fitted a and b values are 1.04 ± 0.05 and 0.04 ± 0.03, respectively. When the fitted expression of V as a function of $2K_{\mathrm{HL}}$ is further introduced into Eq. (S15), ΔE_{S1T1} can be simplified as a function of only $2K_{\mathrm{HL}}$ and $\Delta E_{\mathrm{LUMO-LUMO+1}}$, *i.e.*:

$$\Delta E_{\mathrm{S1T1}}[2K_{\mathrm{HL}}, \Delta E_{\mathrm{LUMO-LUMO+1}}] = 2K_{\mathrm{HL}} - \frac{2((a\times 2K_{\mathrm{HL}})+b)^{2}}{\sqrt{\Delta E_{\mathrm{LUMO-LUMO+1}}{}^{2} + 4((a\times 2K_{\mathrm{HL}})+b)^{2}} + \Delta E_{\mathrm{LUMO-LUMO+1}}} \quad \text{(S17)}$$

Single-Crystal Structure. Single-crystal samples were prepared via the liquid-phase diffusion method with a mixed system of hexane and methanol and analyzed using single-crystal X-ray diffraction (XRD) method. Diffraction data were collected on a Rigaku R-AXIS-RAPID diffractometer in ω-scan mode with graphite-monochromator

MoKα radiation. The structure determination was solved with direct methods via the SHELXTL programs and refined with full-matrix least squares on F^2. Crystallographic data for the structure reported in this article has been deposited at the Cambridge Crystallographic Data Centre under deposition number CCDC 2392596 (crystal of ICZ4B); copy of the data can be obtained free of charge via https://www.ccdc.cam.ac.uk/structures/.

Device fabrication and measurement of EL characteristics. OLEDs were fabricated on indium tin oxide (ITO)-coated transparent glass substrates with multiple layers. The ITO glass substrates have a thickness of ca. 100 nm and a sheet resistance of ca. 30 Ω per square and were cleaned with optical detergent, deionized water, acetone, and isopropanol successively and then dried in an oven. Before device fabrication, the substrates were cleaned with ethanol, isopropanol, and deionized water, dried in an oven, and finally exposed to UV ozone for 15 min. All the organic materials and metal layers were thermally evaporated under a vacuum of ca. 10^{-5} Torr. EL performances of all devices were measured using a Keithley 2400 source meter and an absolute external quantum efficiency (EQE) measurement system (Hamamatsu C9910-11&12).

Experimental

Synthesis

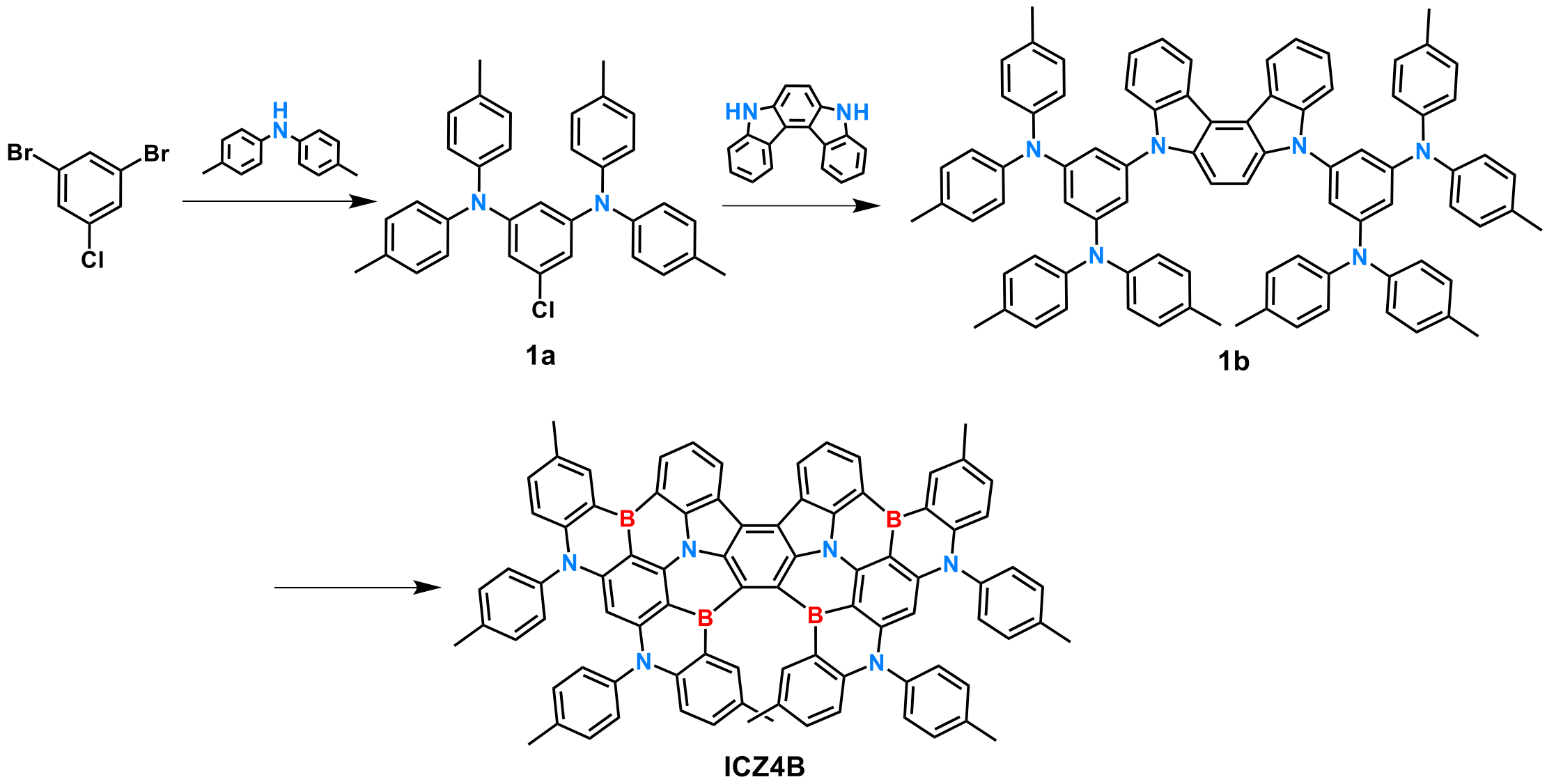

Figure S2 Synthesis route of the target emitter ICZ4B.

Synthesis of 1a

5-chloro-N^1,N^1,N^3,N^3-tetra-p-tolylbenzene-1,3-diamine:

Under a nitrogen atmosphere, a mixture of 1,3-dibromo-5-chlorobenzene (4.1 g, 15 mmol), di-p-tolylamine (7.4 g, 38 mmol), $Pd_2(dba)_3$ (412 mg, 0.5 mmol), s-Phos (380 mg, 0.9 mmol), sodium tert-butoxide (5.8 g, 60 mmol) and toluene (100 mL) were added into a two-necked reaction flask. The mixture was heated to 90 °C and stirred for 8h. After cooling to room temperature, the reaction mixture was quenched with water, extracted with dichloromethane, and the solvent was removed by rotary evaporation. The crude product was further purified by column chromatography to obtain 1a (6.5 g, 85%). ^{1}H NMR (400 MHz, CD_2Cl_2) δ 7.05 (d, J = 8.3 Hz, 8H), 6.95 – 6.90 (m, 8H), 6.45 (t, J = 2.1 Hz, 1H), 6.39 (d, J = 2.0 Hz, 2H), 2.28 (s, 12H). ^{13}C NMR (101 MHz, $CDCl_3$) δ 149.71, 144.67, 134.96, 132.98, 129.98, 124.90, 115.00, 114.13, 20.94. MALDI-TOF: calculated: 502.22, found: 502.20.

Synthesis of 1b

5,5'-(indolo[2,3-c]carbazole-5,8-diyl)bis(N1,N1,N3,N3-tetra-p-tolylbenzene-1,3-

diamine):

Under a nitrogen atmosphere, a mixture of 1a (2.4 g, 6.0 mmol), 5,8-dihydroindolo[2,3-c]carbazole (768 mg, 3.0 mmol), $Pd_2(dba)_3$ (108 mg, 0.12 mmol), s-Phos (98 mg, 0.24 mmol), sodium tert-butoxide (584 mg, 6.0 mmol) and tert-Butylbenzene (30 mL) were added into a two-necked reaction flask. The mixture was heated to 180 °C and stirred for 20 h. After cooling to room temperature, the reaction mixture was quenched with water, extracted with dichloromethane, and the solvent was removed by rotary evaporation. The crude product was further purified by column chromatography to obtain 1b (2.8 g, 78%). ^{1}H NMR (400 MHz, CD_2Cl_2) δ 8.79 (d, *J* = 7.8 Hz, 2H), 7.52 (s, 2H), 7.50 (dd, *J* = 8.2, 1.2 Hz, 2H), 7.47 – 7.41 (m, 2H), 7.36 (ddd, *J* = 8.0, 6.9, 1.3 Hz, 2H), 7.07 (s, 32H), 6.74 (t, *J* = 2.1 Hz, 2H), 6.67 (d, *J* = 2.1 Hz, 4H), 2.27 (s, 24H). ^{13}C NMR (101 MHz, CD_2Cl_2) δ 149.97, 144.72, 140.46, 138.89, 136.14, 133.07, 129.85, 124.88, 124.80, 123.14, 122.60, 119.19, 116.34, 114.04, 113.65, 110.12, 108.94, 20.50. MALDI-TOF: calculated: 1188.58, found: 1188.56.

Synthesis of ICZ4B:

Under a nitrogen atmosphere, BBr_3 (2 mL, 20 mmol) was added dropwise in a solution of 1b (600 mg, 0.5 mmol) in o-dichlorobenzene (15 mL) at room temperature. The mixture was heated to 210 °C and stirred for 24 h. After cooling to room temperature, the reaction mixture was carefully quenched by phosphate buffer and the pH of the reaction mixture was adjusted to 7, extracted with dichloromethane, and the solvent was removed by rotary evaporation. The crude product was purified by column chromatography to obtain ICZ4B (201 mg, 33%). ^{1}H NMR (400 MHz, CD_2Cl_2) δ 9.15 (d, J = 7.6 Hz, 2H), 9.03 (d, J = 7.7 Hz, 2H), 8.88 (s, 2H), 8.07 (s, 2H), 7.86 (d, J = 7.8 Hz, 2H), 7.40 – 7.35 (m, 6H), 7.34 (s, 2H), 7.28 (d, J = 8.1 Hz, 4H), 7.16 (s, 2H), 7.14 – 7.10 (m, 4H), 6.83 – 6.79 (m, 4H), 6.59 (d, J = 8.6 Hz, 2H), 5.54 – 5.53 (m, 2H), 2.56 (s, 12H), 2.54 (s, 6H), 1.86 (s, 6H). The ^{13}C NMR spectrum could not be obtained due to poor solubility. MALDI-TOF: calculated: 1220.53, found: 1220.56.

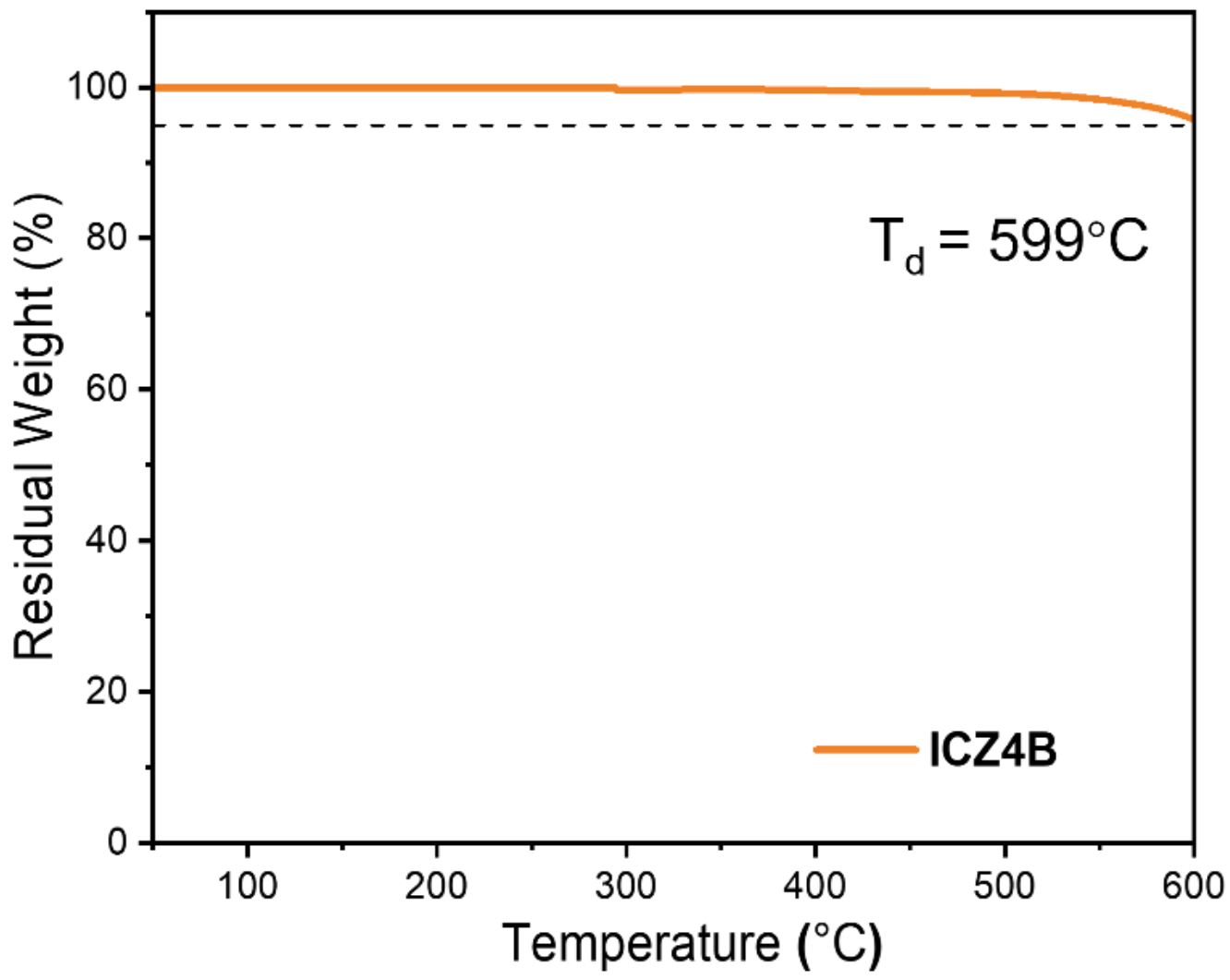

Figure S3. Thermogravimetric analysis curve of ICZ4B.

Table S1. Crystal data and structure refinement for ICZ4B.

Identification code	ICZ4B
Empirical formula	$C_{86}H_{60}B_4N_6$
Formula weight	1220.64
Temperature/K	170
Crystal system	triclinic
Space group	P-1
a/Å	11.6258(16)
b/Å	16.304(2)
c/Å	20.155(2)
α/°	102.230(9)
β/°	97.507(9)
γ/°	99.479(9)
Volume/Å^3	3628.0(8)
Z	2
ρ_{calc}g/cm^3	1.117
μ/mm^{-1}	0.495
F(000)	1276.0
Crystal size/mm^3	0.11 × 0.05 × 0.04
Radiation	CuKα (λ = 1.54178)
2Θ range for data collection/°	4.554 to 127.496
Index ranges	$-13 \le h \le 13$, $-18 \le k \le 18$, $-23 \le l \le 20$
Reflections collected	25622
Independent reflections	11594 [R_{int} = 0.0887, R_{sigma} = 0.1151]
Data/restraints/parameters	11594/0/873

Goodness-of-fit on F^2	1.091
Final R indexes [I>=2σ (I)]	R_1 = 0.1070, wR_2 = 0.2858
Final R indexes [all data]	R_1 = 0.1635, wR_2 = 0.3283
Largest diff. peak/hole / e Å^{-3}	0.29/-0.33

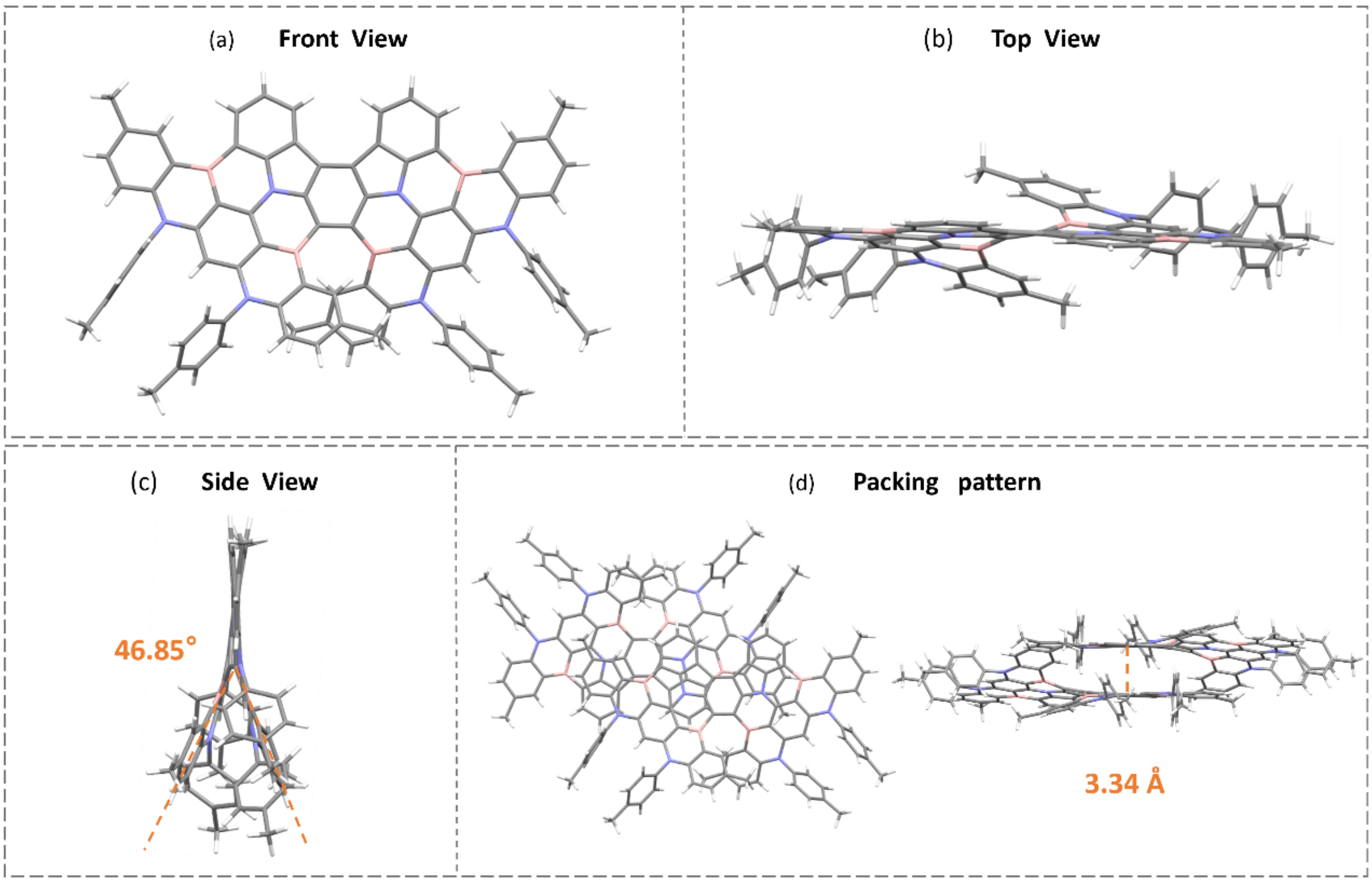

Figure S4. Molecular geometries of ICZ4B determined from single-crystal X-ray diffraction analysis in (a) front view and (b) side view (dihedral angle of ortho-fragments) and (c) packing mode with highlighted intermolecular plane–plane distance.

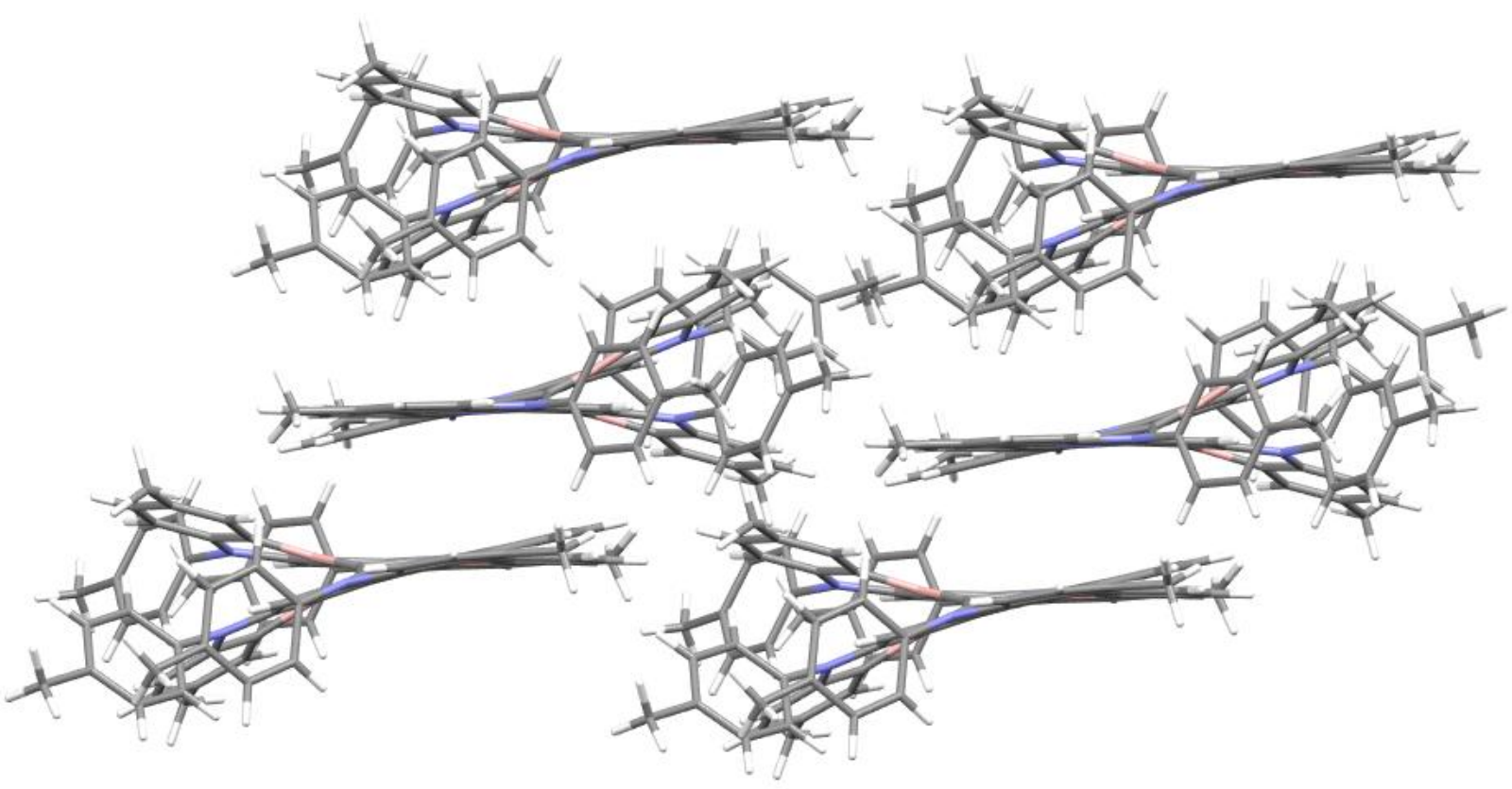

Figure S5. Molecular packing mode with more ICZ4B molecules.

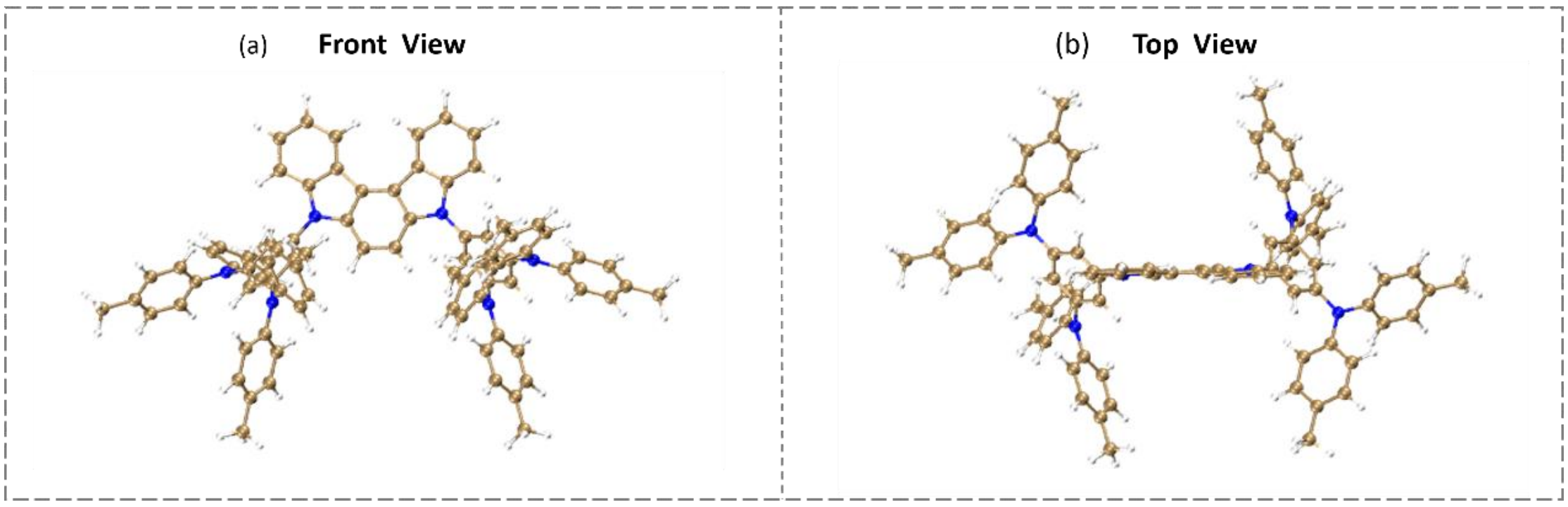

Figure S6. Optimized geometry of the ground state of ICZ4MN based on the DFT calculation in (a) front view and (b) top view.

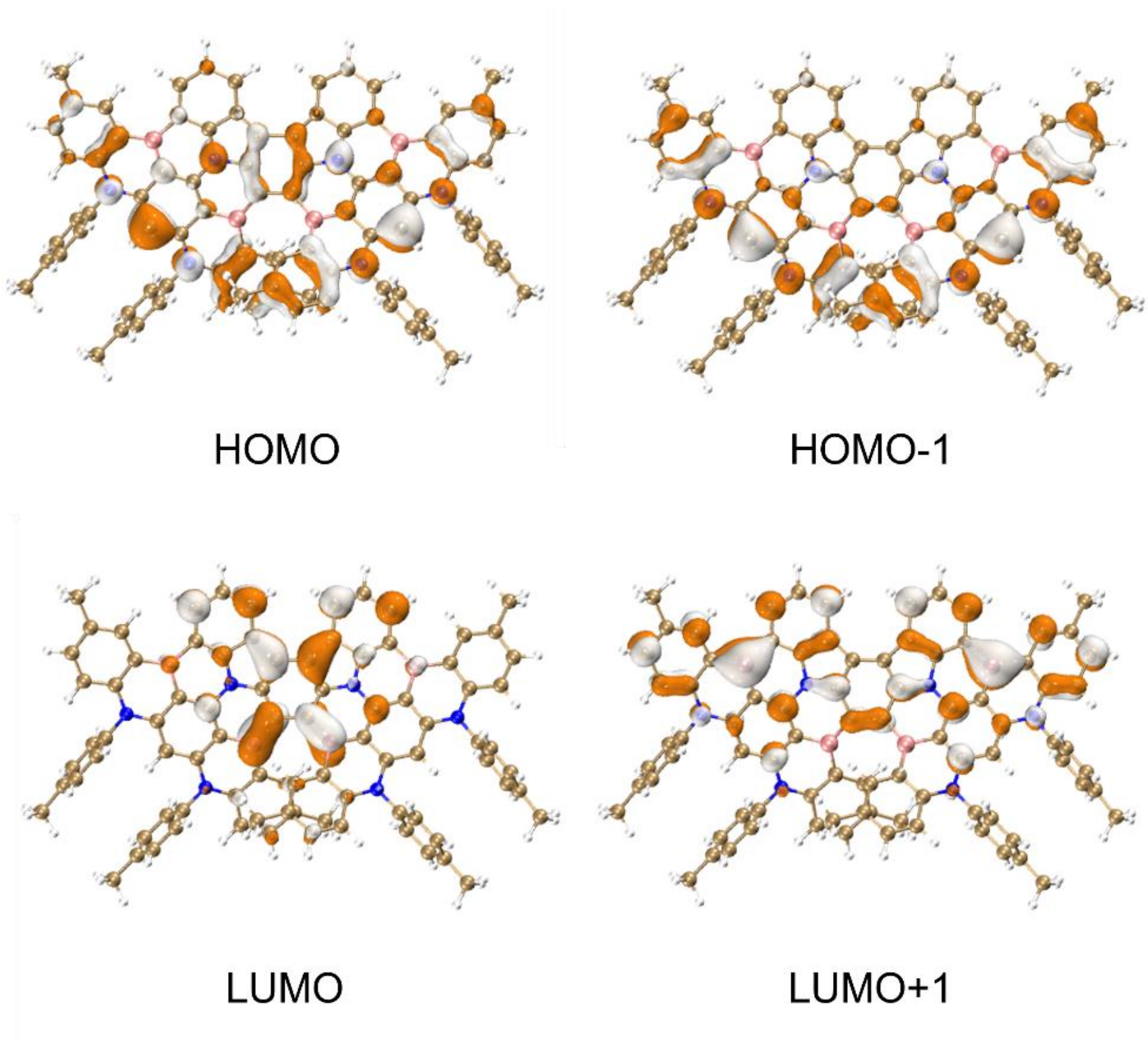

Figure S7. Distributions of the lowest unoccupied molecular orbital (LUMO), LUMO+1, highest occupied molecular orbital (HOMO) and HOMO−1 of ICZ4B.

Table S2. Summary of calculated energy levels of FMOs and energy gaps.

Emitters	HOMO−1 (eV)	HOMO (eV)	LUMO (eV)	LUMO+1 (eV)	E_g (eV)
ICZ4B	−4.75	−4.58	−1.81	−1.20	2.77

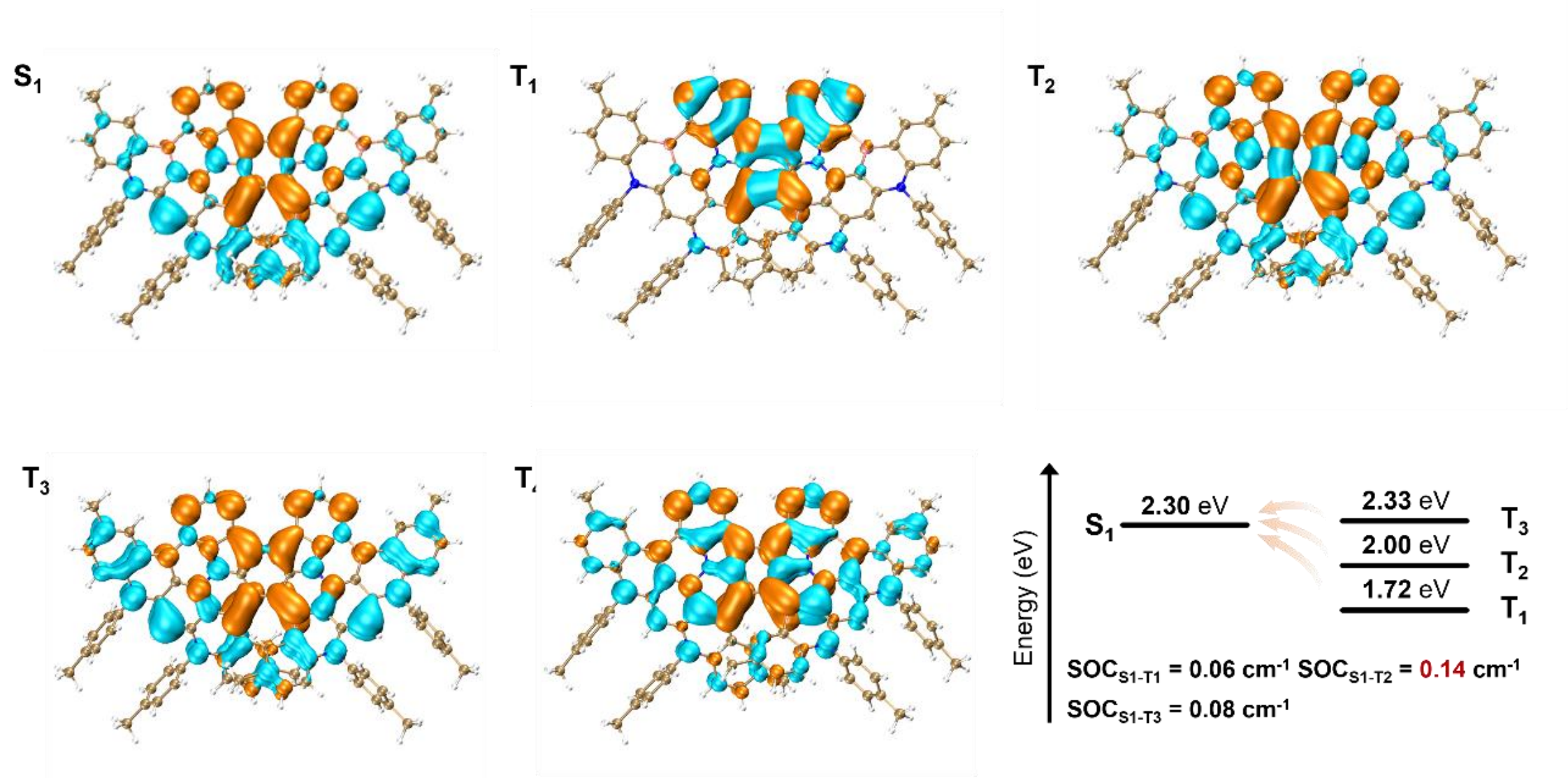

Figure S8. Calculated difference-density plots of the excited states (hole/electron wavefunctions shown in blue/orange) and energy level alignments and SOC matrix elements of ICZ4B.

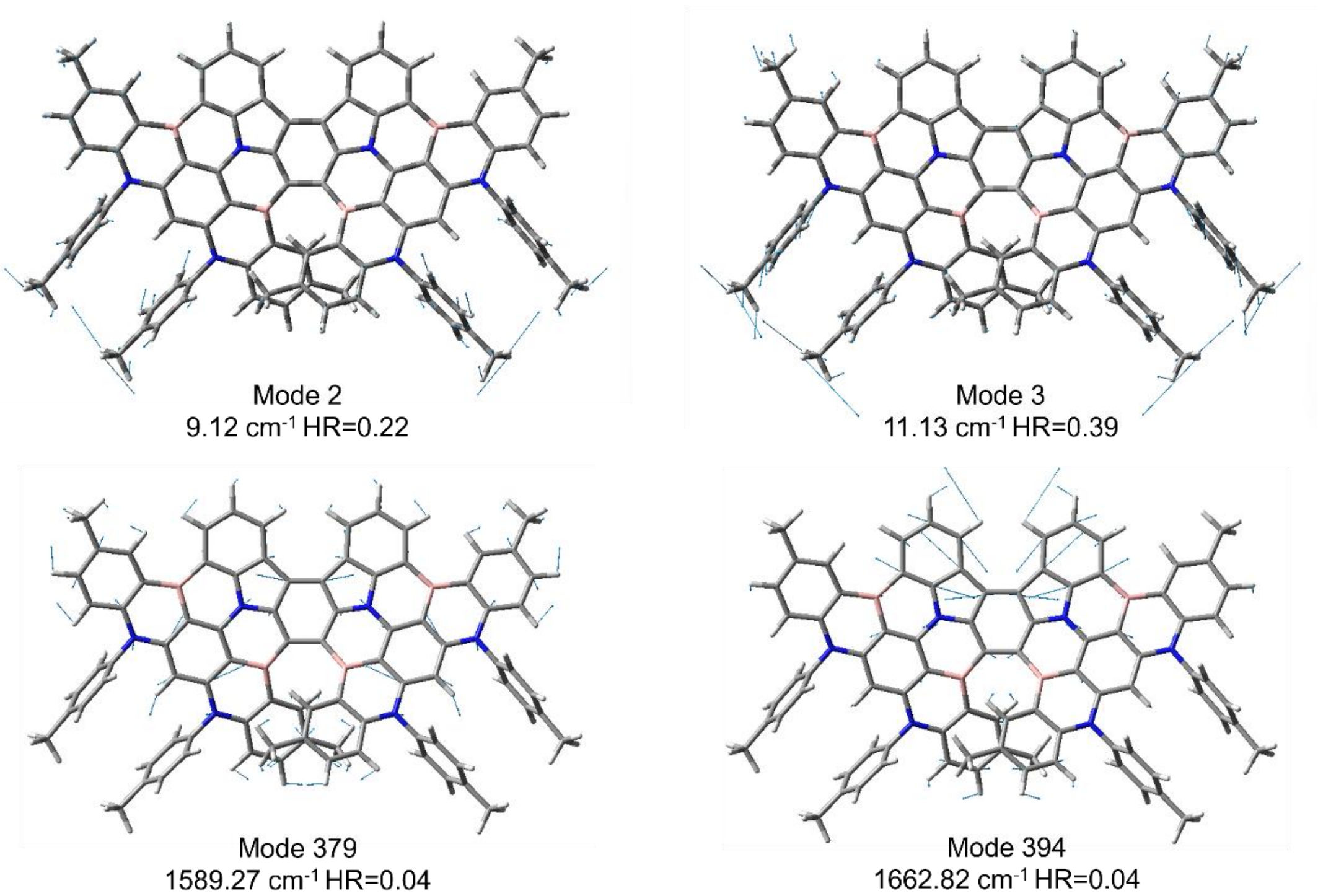

Figure S9. Representative vibration modes in the S1→S0 transition and the corresponding Huang-Rhys (HR) factors.

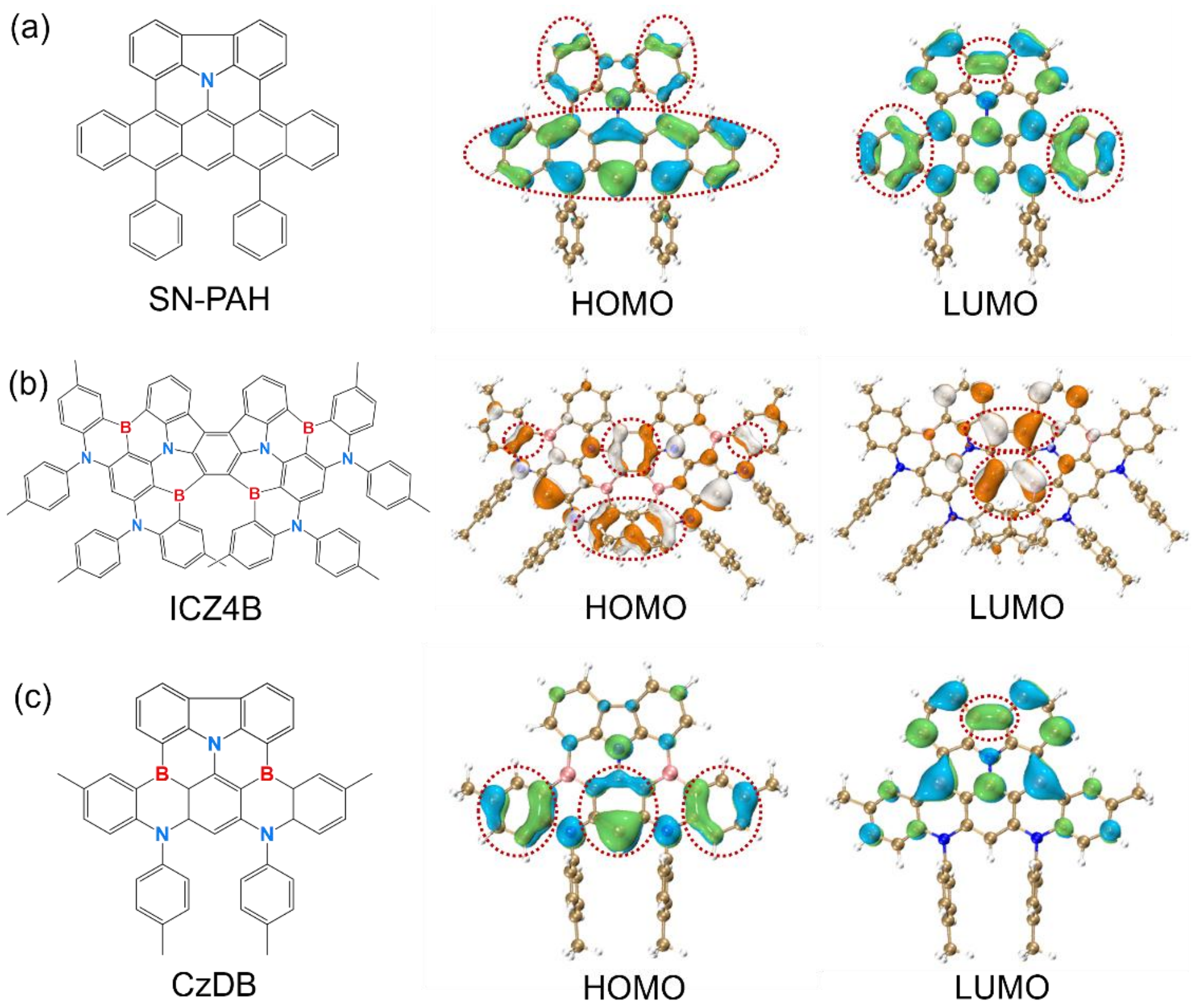

Figure S10. The FMO distributions of (a) SN-PAH, (b) ICZ4B and (c) CzDB (those with clear bonding characters are indicated by the red dot circles).

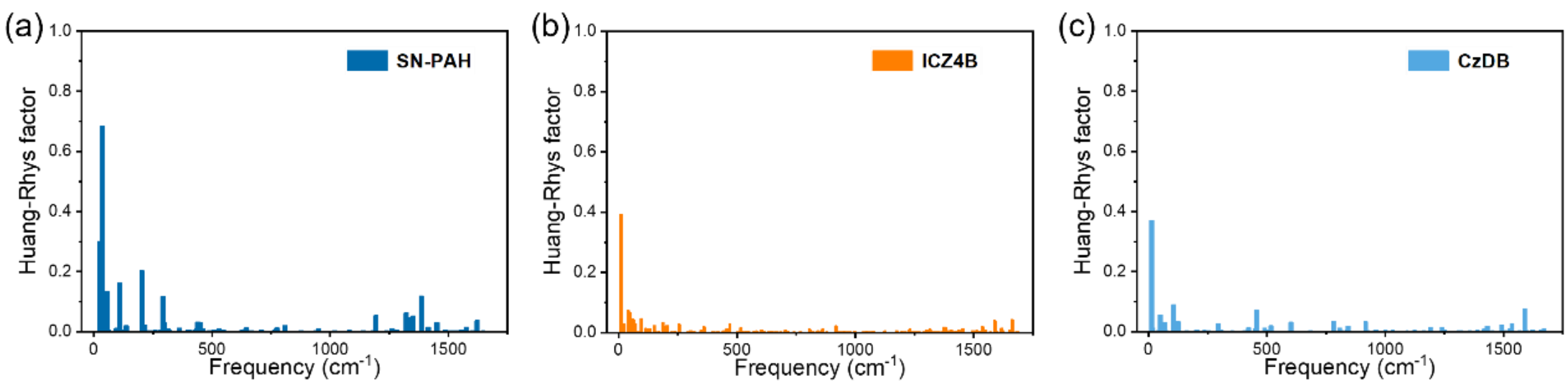

Figure S11. Calculated HR factor for (a) SN-PAH, (b) ICZ4B and (c) CzDB.

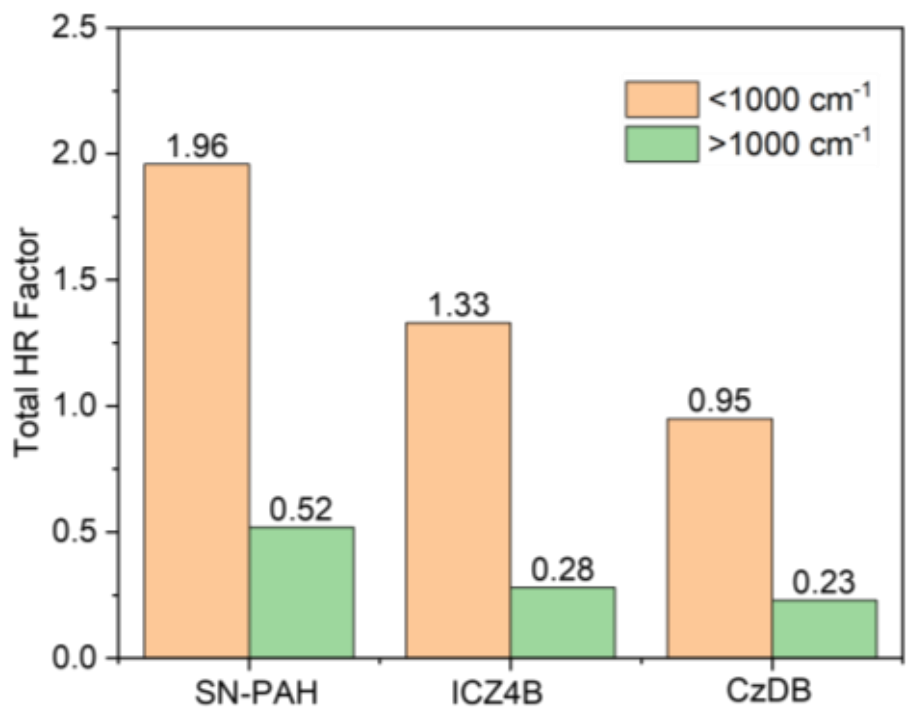

Figure S12. Total HR factor for SN-PAH, ICZ4B and CzDB.

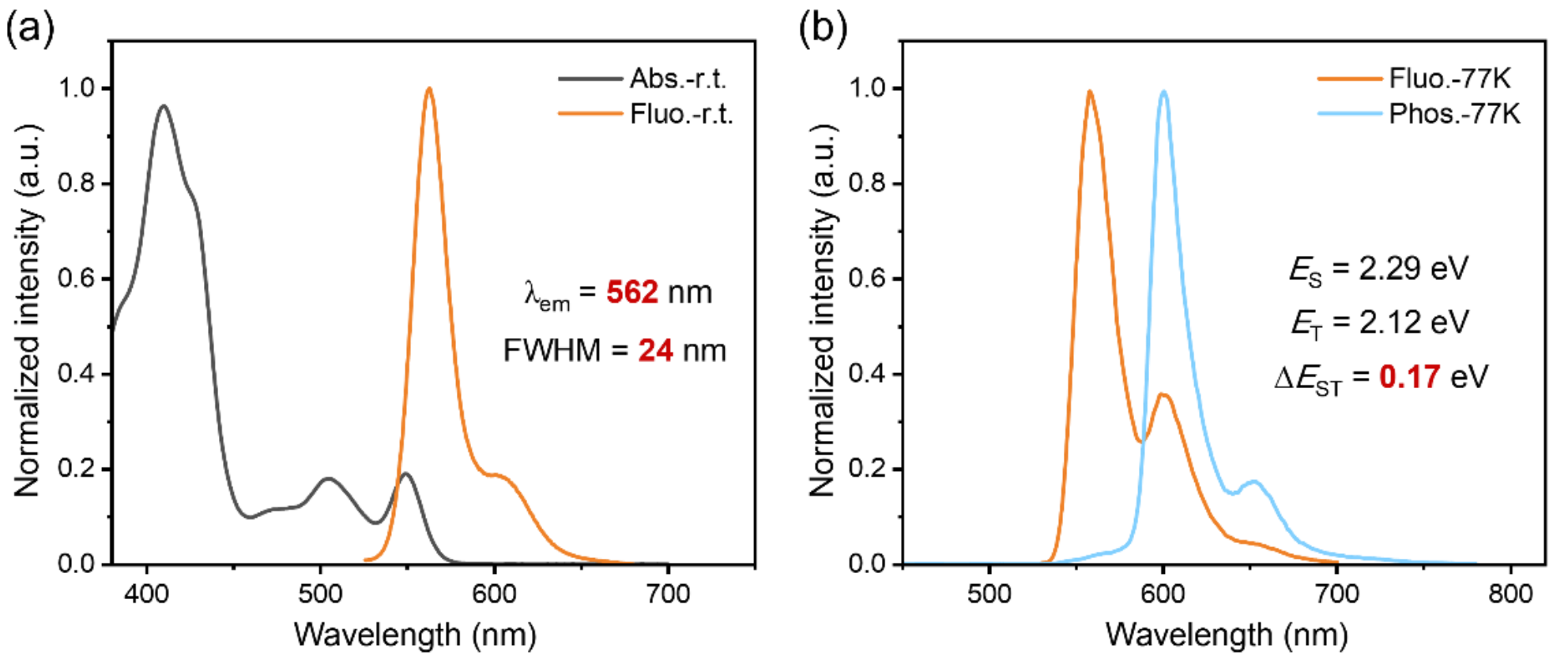

Figure S13. (a) Ultraviolet–visible absorption, fluorescence spectra at room temperature and (b) fluorescence and phosphorescence spectra at 77 K in dilute toluene.

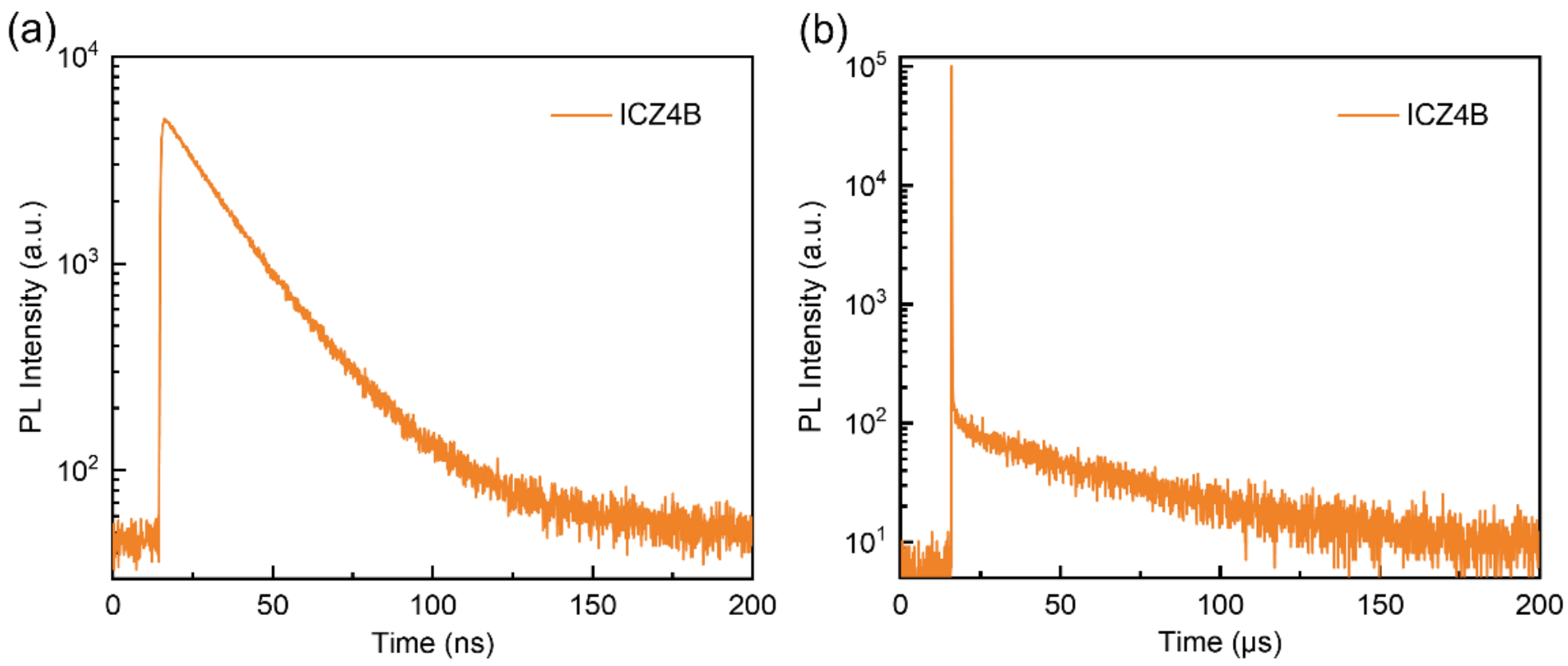

Figure S14. Transient PL decay spectra for (a) prompt decay and (b) delayed decay curves of 1 wt% ICZ4B doped in SF_3-TRZ film.

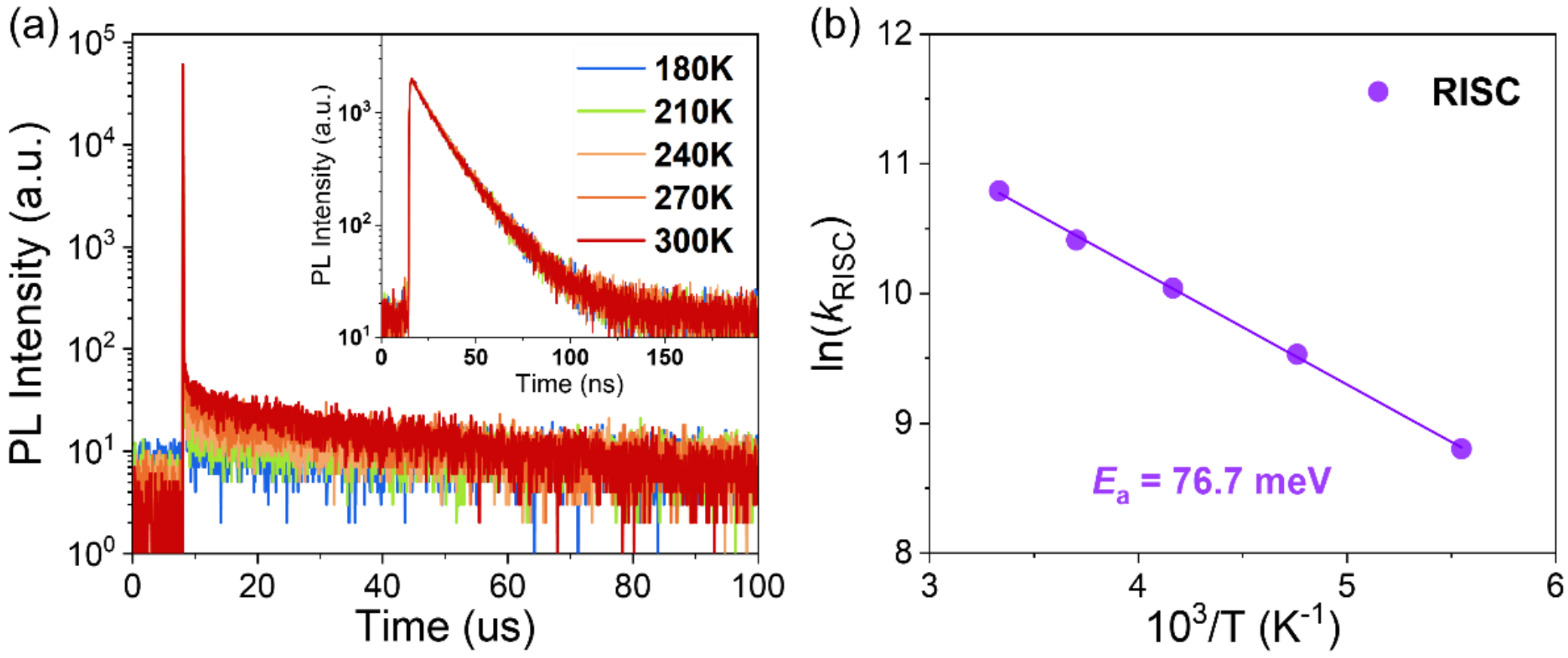

Figure S15. (a) Temperature-dependent transient PL decay curves and (b) Arrhenius analysis of delayed component of 1 wt% ICZ4B doped in SF_3-TRZ film.

Table S3. Summary of kinetic constant data of ICZ4B (1 wt% doped in SF_3-TRZ film) at 300 K.

	Φ_{PL}[a] (%)	Φ_p/Φ_d [a]	τ_p[c] (ns)	τ_d[d] (μs)	k_{ISC}[e] (10^7 s^{-1})	k_{RISC}[f] (10^4 s^{-1})	k_r [g] (10^7 s^{-1})	k_{nr} [h] (10^5 s^{-1})
ICZ4B	99	68/31	20.9	30.7	1.5	4.7	3.2	3.3

[a] PLQY determined from 1 wt%-doped films in SF_3-TRZ at 300 K under a N_2 atmosphere. [b] Fluorescence quantum yields for prompt/delayed fluorescence. [c] Lifetime of prompt fluorescence component. [d] Lifetime of delayed fluorescence component. [e] The rate constant of intersystem crossing ($S_1 \rightarrow T_1$). [f] The rate constant of reverse intersystem crossing ($T_1 \rightarrow S_1$). [g] The rate constant of fluorescence radiative decay ($S_1 \rightarrow S_0$). [h] The rate constant of non-radiative decay.

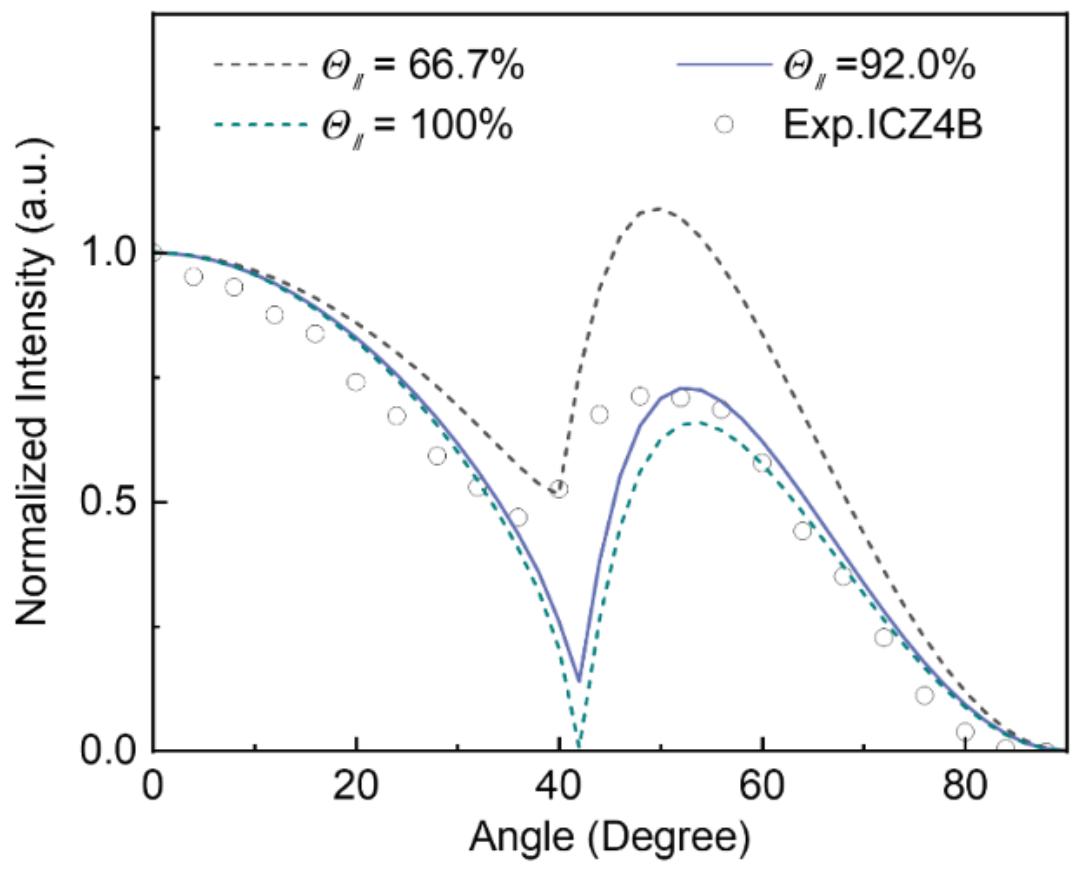

Figure S16. The horizontal molecular orientation ratio in the 1 wt%-doped film of ICZ4B.

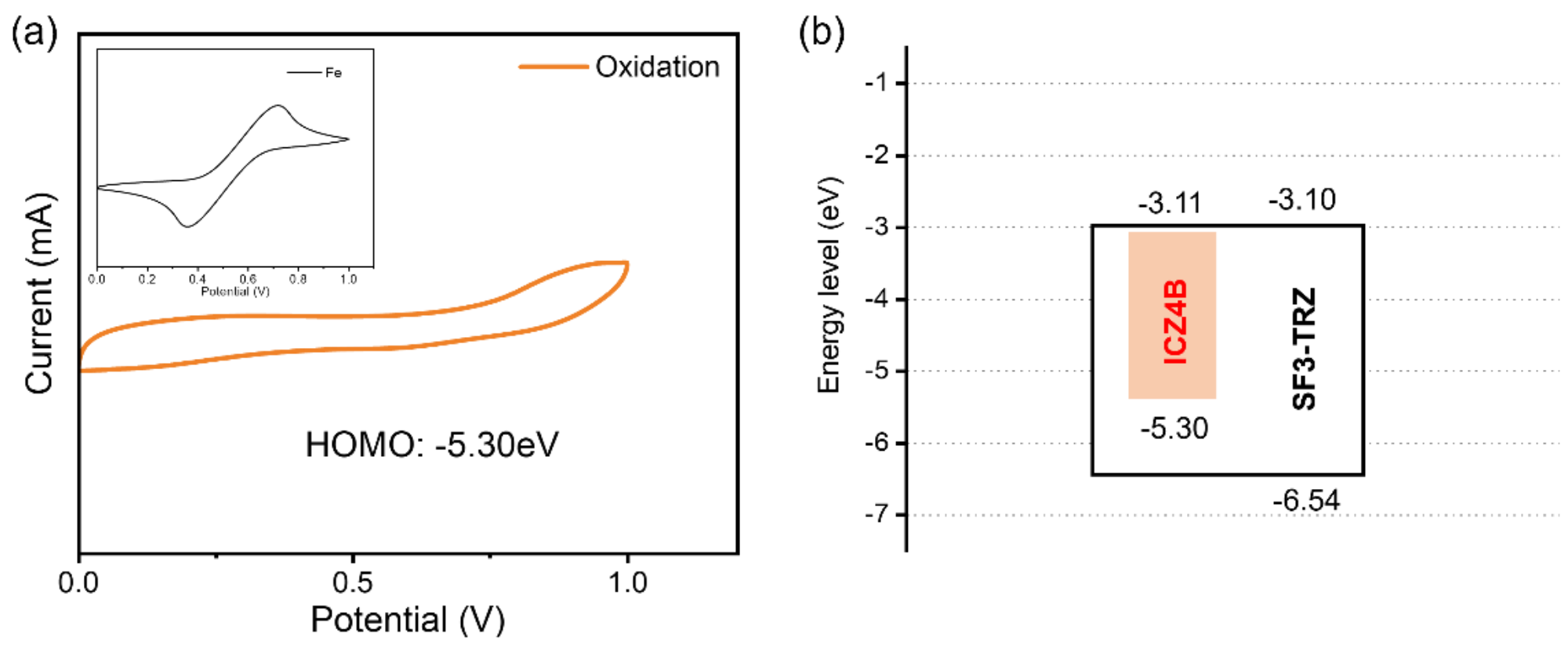

Figure S17. (a) Cyclic voltammetry curve of ICZ4B and (b) energy levels of ICZ4B and SF_3-TRZ.

Table S4. Summary of the ΔE_{ST} and k_{RISC} from photoluminescence (PL) spectrum of reported long-wavelength MR emitters.

MR emitters	λ_{PL} [nm]	ΔE_{ST} [eV]	k_{RISC} [10^4 s^{-1}]	Ref
ICZ4B	562	0.17	4.7	This work
DACz-B	572	0.14	1.0	*Angew. Chem. Int. Ed.* **2021**, *60*, 23142.
DB2	564	0.32	/	*Adv. Funct. Mater.* **2024**, 34, 2316323.
DB3	606	0.26	/	
BN-R	624	0.11	1.1	*Angew. Chem. Int. Ed.* **2023**, 62, e202216473.
BBCz-R	615	0.19	1.2	*J. Am. Chem. Soc.* **2020**, 142, 46, 19468–19472.
R-BN	662	0.18	6.7	*Angew. Chem. Int. Ed.* **2021**, *60*, 20498.
R-TBN	692	0.16	2.5	
BNO1	605	0.25	/	*Adv. Mater.* **2022**, 34, 2201442.
BNO2	609	0.27	/	
BNO3	616	0.26	/	
B4N6-Me	580	0.19	0.8	*Angew. Chem. Int. Ed.* **2023**, 62, e202305580.
2TXBN	582	0.15	0.5	*Angew. Chem. Int. Ed.* **2025**, 64, e202423602.
BNTXBN	585	0.25	0.2	
HBN	572	0.22	2.5	*Angew. Chem. Int. Ed.* **2025**, 64, e202421102.
BNTPA	613	0.10	16	*J. Am. Chem. Soc.* **2024**, 146, 47, 32826–32836.
Δ-DABNA-TB	613	/	0.1	*J. Am. Chem. Soc.* **2024**, 146, 27, 18331–18340.
BN-IDP	604	0.39	/	*Chem. Eng. J.* **2025**, 520, 166027
BN-nap3	564	0.31	/	*Adv. Mater.* **2026**, e13180.
CMBNO	604	0.26	2.4	*Adv. Opt. Mater.* **2025**, e01766.
TPBNO	609	0.25	9.3	
*t*DPABNO	625	0.21	1.3	
RBNO2	645	0.26	/	*Mater. Horiz.,* **2023**, 10, 3712-3718

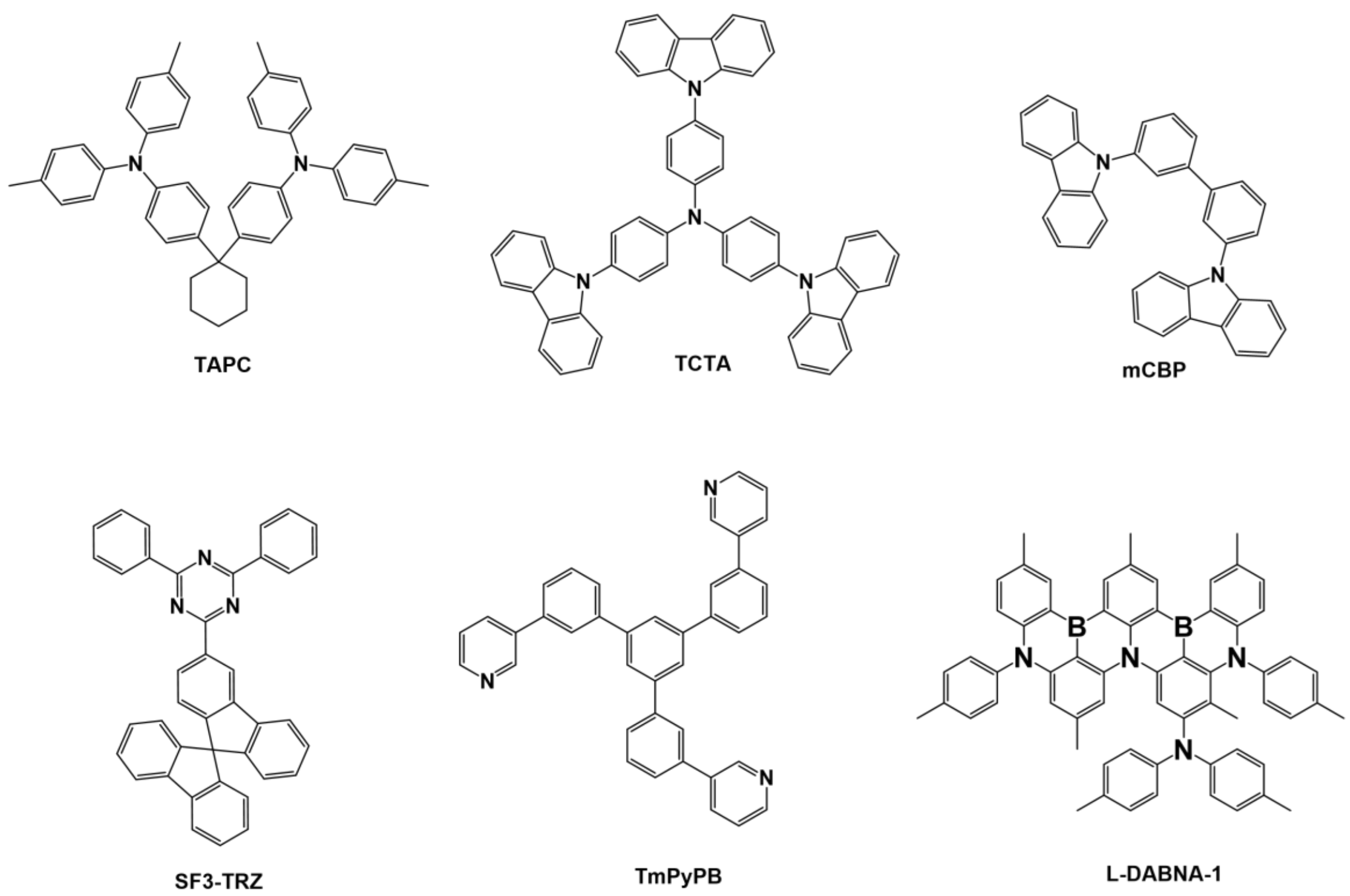

Figure S18. The chemical structures of the materials used in the devices.

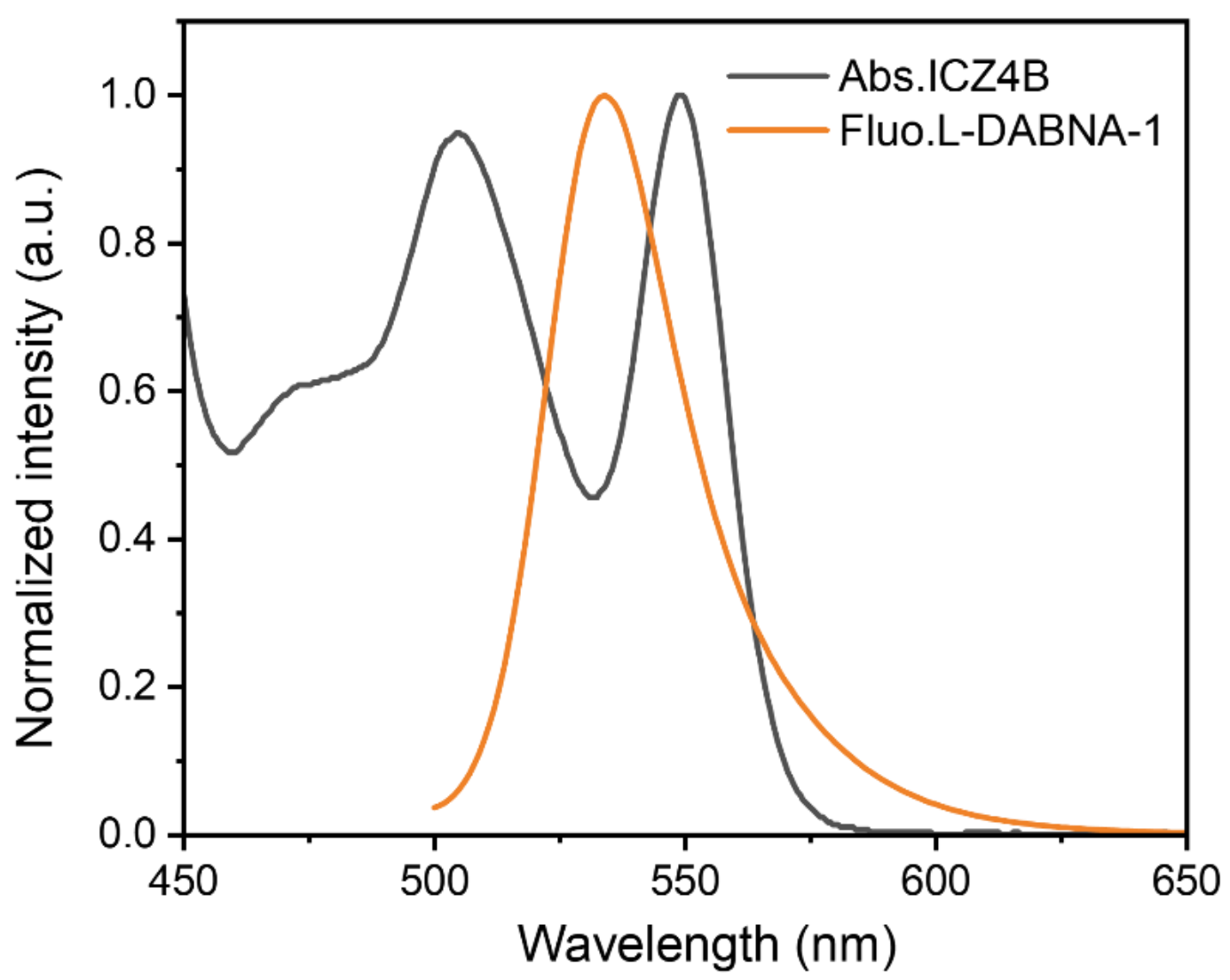

Figure S19. Ultraviolet–visible absorption of ICZ4B and fluorescence spectra of L-DABNA-1 at room temperature.

Table S5. Summary of EL performance data of ICZ4B with different doping concentrations.

Devices[a]	λ_{EL}[a] [nm]	FWHM[a] [nm]	$EQE_{max/100/1000}$[b] [%]	CE_{max}[c] [cd A^{-1}]	PE_{max}[c] [lm W^{-1}]	CIE[a] (x, y)
0.5 wt%	568	32	33.9/14.4/4.3	122.7	107.0	(0.47, 0.52)
1 wt%	568	33	36.5/26.0/9.4	133.0	116.0	(0.48, 0.52)
2 wt%	568	33	34.5/27.1/9.7	124.0	108.0	(0.48, 0.51)
TSF 1 wt%	568	36	33.7/31.0/17.4	114.2	105.5	(0.46, 0.53)

[a] EL spectra at 100 cd m^{-2}. [b] The maximum EQEs and EQEs at 100 and 1000 cd m^{-2}. [c] The maximum CE and PE.

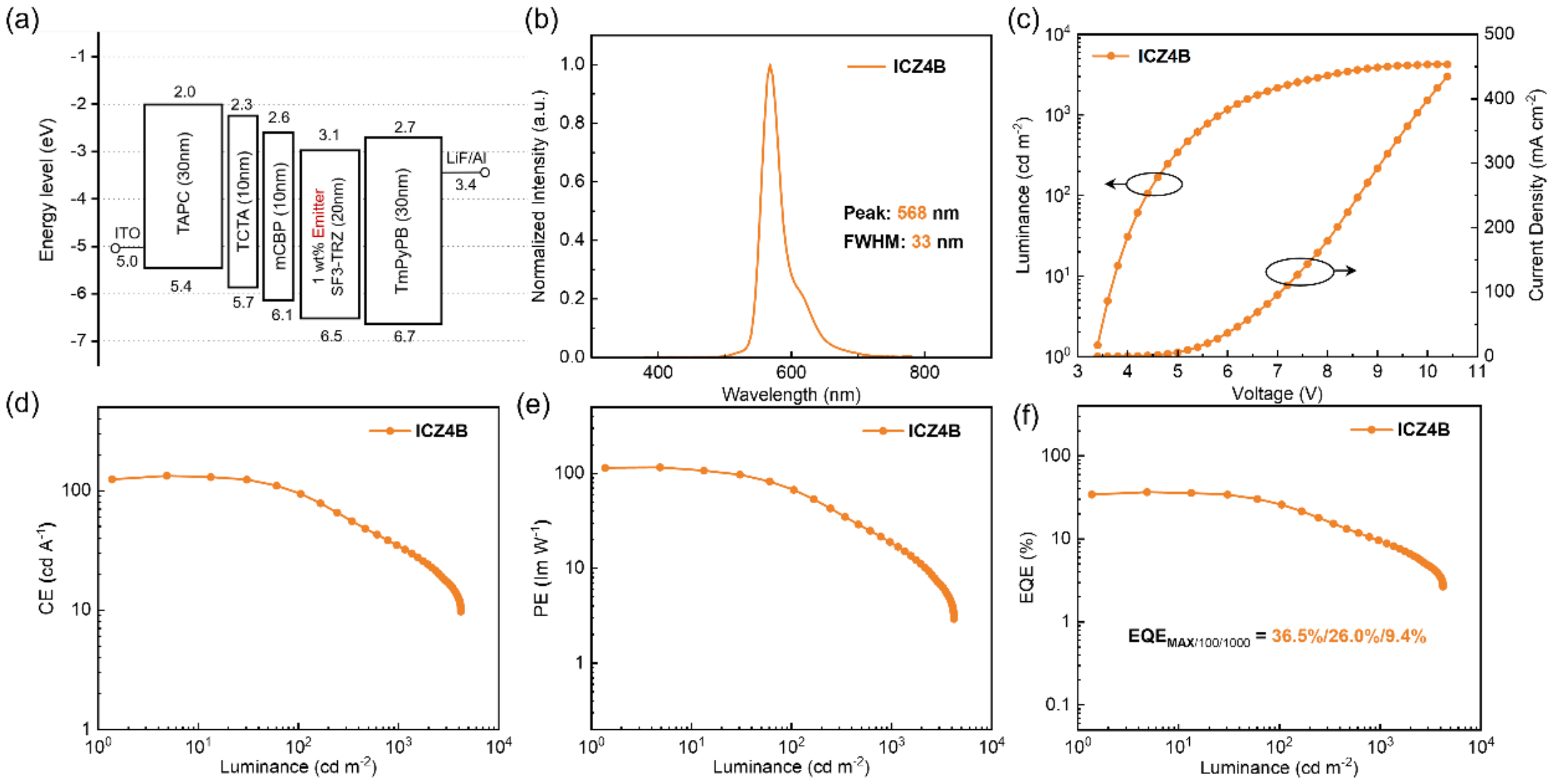

Figure S20. (a) Device structure, (b) Electroluminescence spectrum, (c) Luminance-Voltage-Current Density curves, (d) Current efficiency-Luminance and (e) Power efficiency-Luminance curves and (f) EQE-Luminance curve of 1 wt% ICZ4B doped device.

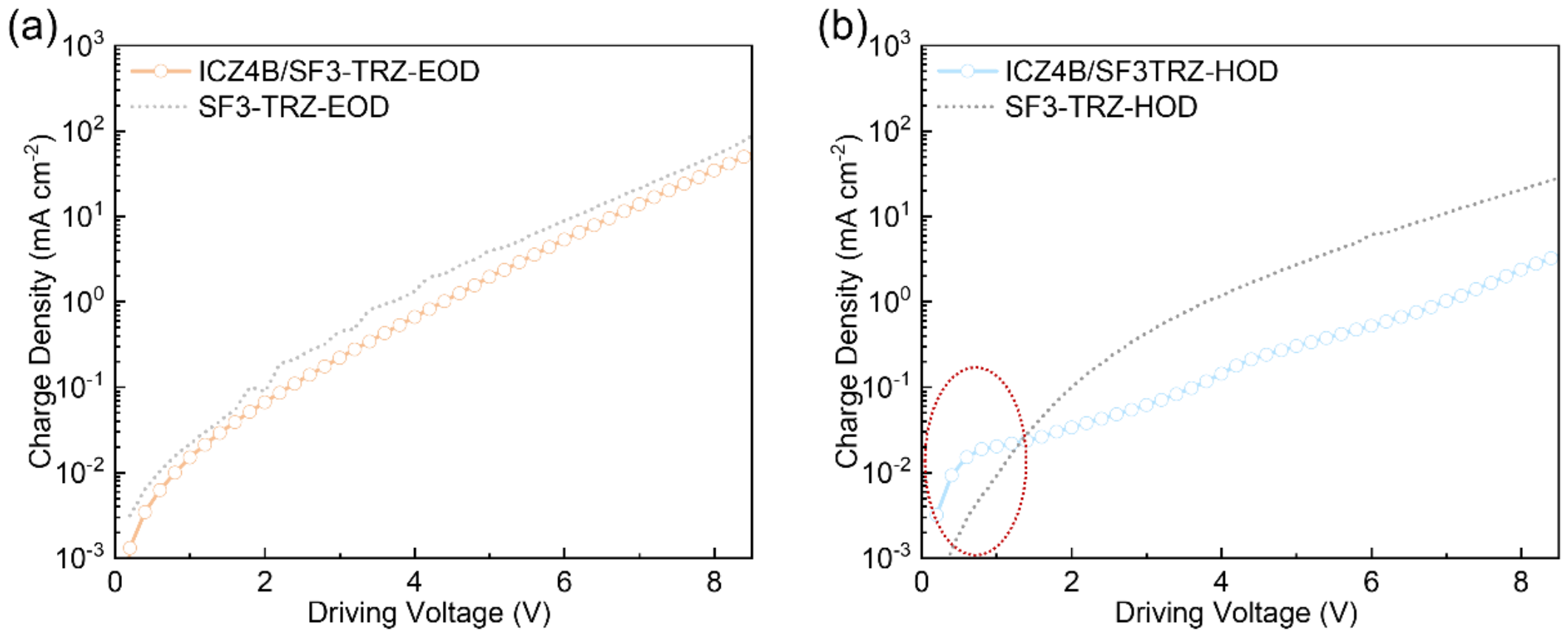

Figure S21. Current density–Voltage characteristics of (a) electron-only devices (EODs) and (b) hole-only devices (HODs) (EODs: ITO/ Al (40 nm)/ LiF (1 nm)/ SF_3-TRZ: 1 wt % ICZ4B (50 nm)/ LiF (1 nm)/ Al (100 nm); HODs: ITO/ MoO_3 (12 nm)/ SF_3-TRZ: 1 wt % ICZ4B (50 nm)/ MoO_3 (12 nm)/ Al (100 nm)).

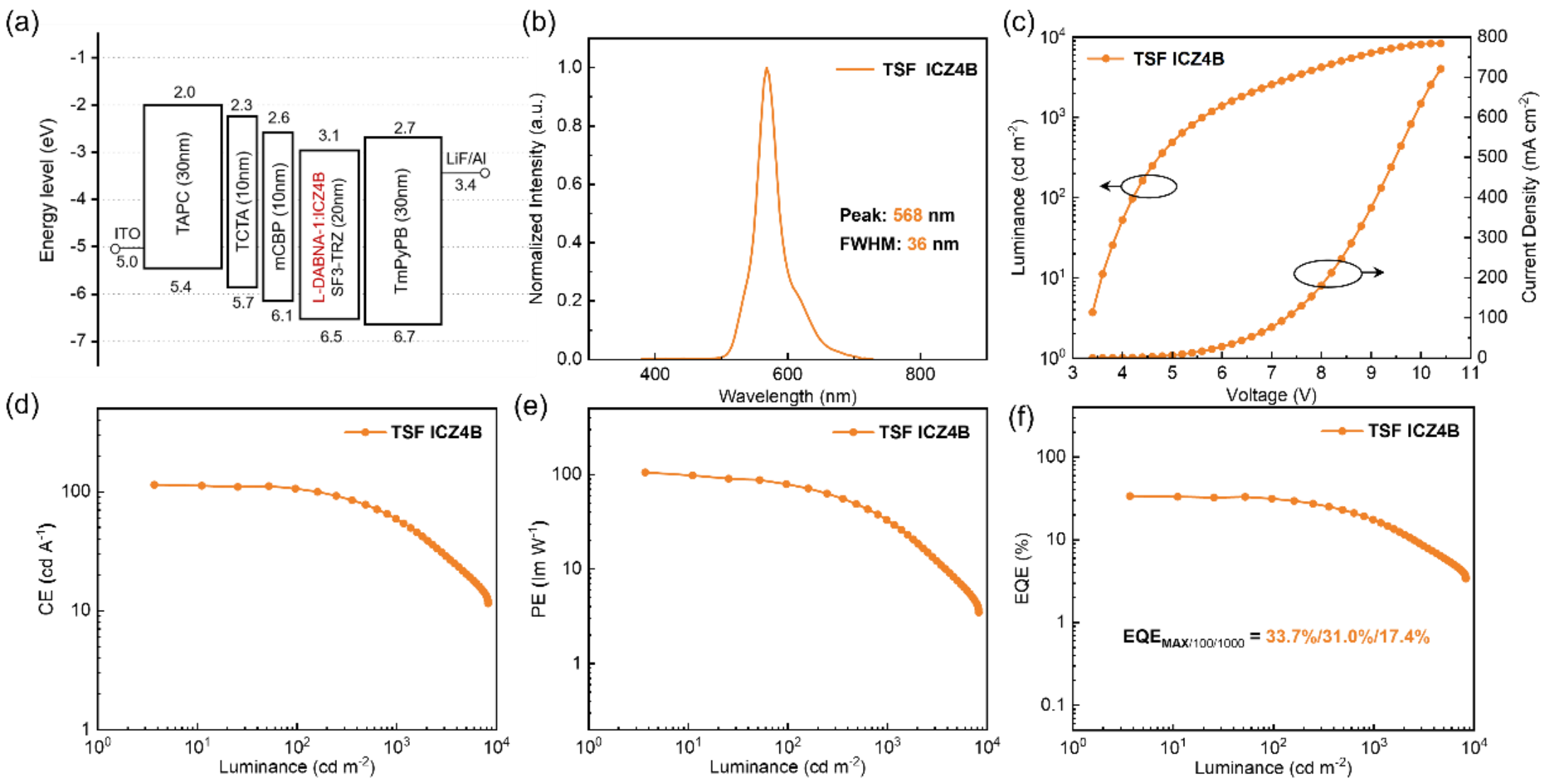

Figure S22. (a) Device structure, (b) Electroluminescence spectrum, (c) Luminance-Voltage-Current Density curves, (d) Current efficiency-Luminance and (e) Power efficiency-Luminance curves and (f) EQE-Luminance curve of 4 wt% L-DABNA-1: 1 wt% ICZ4B doped device.

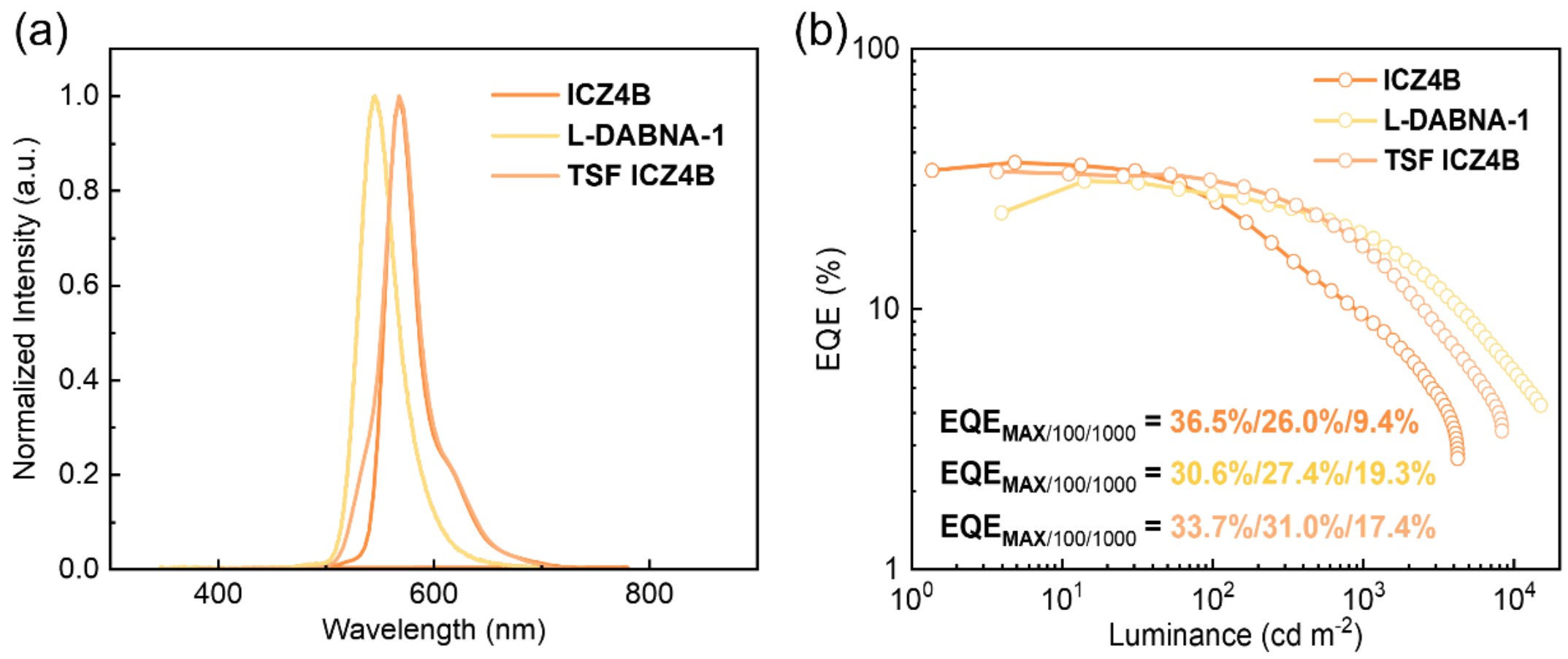

Figure S23. (a) Electroluminescence spectra and (b) EQE-Luminance curves of 1 wt% ICZ4B, 4 wt% L-DABNA-1 and 4 wt% L-DABNA-1: 1 wt% ICZ4B doped devices.

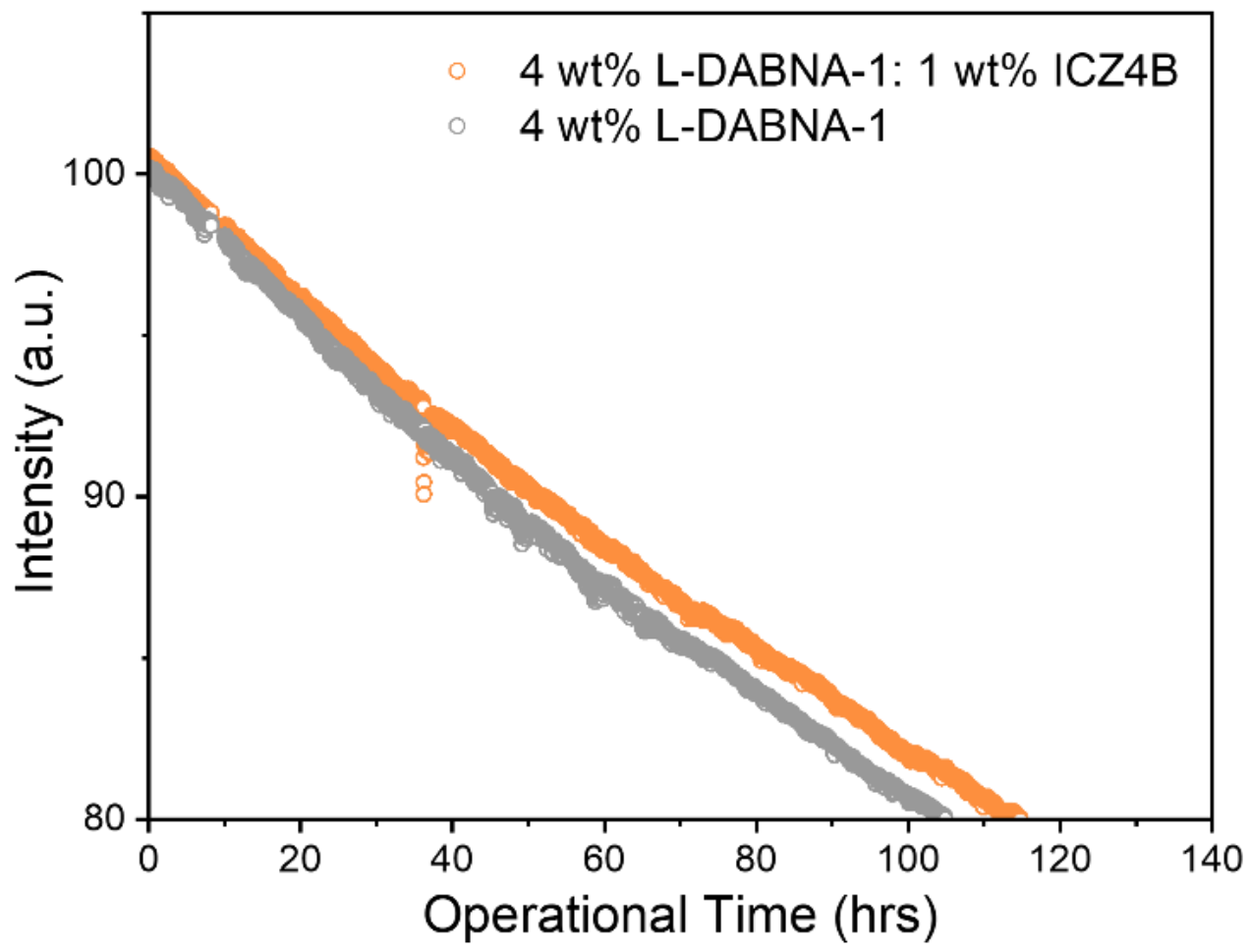

Figure S24. Operation lifetime of the devices of 4 wt% L-DABNA-1 and 4 wt% L-DABNA-1: 1 wt% ICZ4B measured at an initial luminance of 1000 cd m^{-2}. (device structure: ITO/ HAT-CN (10 nm)/ TrisPCz (30 nm)/ mCBP (5 nm)/ EML (30 nm)/ SF_3-TRZ (10 nm)/ SF3-TRZ: 40 wt% Liq (40 nm)/ Liq (2 nm)/ Al (100 nm)). The estimated LT_{80} at the initial luminance of 1000 cd m^{-2} is 104.9 and 114.8 h.

Table S6. Summary of binary devices performance of reported long-wavelength MR emitters with emission maxima beyond 560 nm.

MR emitters	λ_{PL}[a] [V]	FWHM[a] [nm]/[meV]	λ_{EL}[b] [nm]	FWHM[b] [nm]/[meV]	EQE_{max}[c] [%]	Ref
ICZ4B	562	24/93	568	33/126	36.5	This work
DACz-B	572	34/130	571	44/170	19.6	*Angew. Chem. Int. Ed.* **2021**, *60*, 23142.
BN-R	624	46/150	617	47/150	22.0	*Angew. Chem. Int. Ed.* **2023**, 62, e202216473.
BBCz-R	615	21/70	616	26/-	22.0	*J. Am. Chem. Soc.* **2020**, 142, 46, 19468–19472.
R-BN	662	38/-	664	48/-	25.6	*Angew. Chem. Int. Ed.* **2021**, *60*, 20498.
R-TBN	692	38/-	686	48/-	24.7	
BNO1	605	32/-	609	37/-	14.9	*Adv. Mater.* **2022**, 34, 2201442.
BNO2	609	32/-	623	39/-	12.0	
BNO3	616	33/-	616	38/-	15.1	
DB2	564	40/-	573	41	9.1	*Adv. Funct. Mater.* **2024**, 34, 2316323.
DB3	606	35/-	606	35	17.8	
RBNO2	645	39/110	645	49/-	15.1	*Mater. Horiz.*, **2023**, 10, 3712-3718
B4N6-Me	580	19/70	588	27/99	31.7	*Angew. Chem. Int. Ed.* **2023**, 62, e202305580.
HBN	572	17/64	580	24/-	26.7	*Angew. Chem. Int. Ed.* **2025**, 64, e202421102.
BNTPA	613	42/140	617	48/150	35.2	*J. Am. Chem. Soc.* **2024**, 146, 47, 32826–32836.
Δ-DABNA-TB	613	31/100	624	42/-	23.3	*J. Am. Chem. Soc.* **2024**, 146, 27, 18331–18340.
BN-nap3	564	34/130	-/-	-/-	24.5	*Adv. Mater.* **2026**, e13180.

BN-R1	608	43/-	610	44/-	20.7	*Adv. Opt. Mater.* **2024**, 12, 2302811.
BN-R2	595	36/-	601	40/-	21.1	
BN-R3	585	40/-	585	43/-	19.9	
α-NAICZ-BN	605	26/-	615	33/-	18.7	*J. Am. Chem. Soc.* **2026,** 148, 10, 10744–10752.

[a] Peak wavelength and FWHM of the photoluminescence (PL) spectrum. [b] Peak wavelength and FWHM of the electroluminescence (EL) spectrum. [c] Maximum external quantum efficiency.

Table S7. Cartesian coordinates of ICZ4MN at the optimized S_0 geometry.

C	-1.36432	0.125621	-2.118533	C	4.867851	-1.102493	4.933518
C	-0.689719	0.068857	-0.890163	C	4.501595	-1.285138	3.602223
C	0.689578	-0.069387	-0.890156	C	-12.087427	0.029688	1.461163
C	1.364133	-0.126723	-2.118523	C	-5.665707	-7.039516	-1.249103
C	0.70866	-0.042038	-3.375078	C	-6.037458	1.848024	7.059609
C	-0.70889	0.040337	-3.375071	C	12.087198	-0.028452	1.461402
N	-2.746865	0.215808	-2.318363	C	6.037314	-1.844571	7.060295
C	-2.995084	0.16206	-3.685193	C	2.918359	-7.834093	-0.221992
N	2.746671	-0.216991	-2.318359	C	-2.916506	7.833506	-0.224932
C	2.994843	-0.163888	-3.685222	C	5.665139	7.039129	-1.251798
C	-1.757812	0.039778	-4.379413	H	-1.238517	0.121256	0.043642
C	-1.795272	-0.085033	-5.777701	H	1.23841	-0.121348	0.043653
C	-3.01343	-0.040078	-6.446875	H	-0.885955	-0.246995	-6.341386
C	-4.216054	0.128419	-5.740337	H	-3.03396	-0.139812	-7.52852
C	-4.222047	0.227121	-4.353206	H	-5.157464	0.172611	-6.281509
C	4.221786	-0.229244	-4.353241	H	-5.149676	0.342112	-3.802463
C	4.215747	-0.131197	-5.740418	H	5.149434	-0.34396	-3.802472
C	3.013098	0.036947	-6.446997	H	5.157141	-0.17563	-6.2816
C	1.79496	0.082202	-5.777805	H	3.033592	0.136167	-7.52869
C	1.757546	-0.041945	-4.379457	H	0.885624	0.243885	-6.341539
C	-3.730733	0.329013	-1.295139	H	-4.756127	-1.464729	-1.896518
C	3.730575	-0.329692	-1.295114	H	-6.4036	0.638548	1.481682
C	-4.736656	-0.635177	-1.200401	H	-2.889228	2.14385	-0.501524
C	-5.709978	-0.524594	-0.19353	H	4.755834	1.463853	-1.897302
C	-5.654991	0.551695	0.703862	H	6.403535	-0.637832	1.481776
C	-4.642389	1.516915	0.606415	H	2.889217	-2.144241	-0.500669
C	-3.673284	1.402902	-0.403857	H	-8.30574	-2.73571	1.687243
N	-6.740726	-1.490253	-0.088068	H	-10.592375	-2.08923	2.347087
N	-4.594709	2.596336	1.522808	H	-10.401764	1.287421	-0.297047
C	4.736441	0.634603	-1.200823	H	-8.128432	0.624962	-0.981558
C	5.709797	0.524531	-0.193934	H	-8.273919	-3.150335	-1.520757

C	5.654903	-0.551367	0.703935	H	-7.832328	-5.530417	-1.988376
C	4.642354	-1.516683	0.606943	H	-4.161466	-5.286206	0.224058
C	3.673218	-1.403187	-0.403354	H	-4.617184	-2.916098	0.721304
N	4.594708	-2.595697	1.523835	H	-6.152901	4.192618	2.998973
N	6.740488	1.49031	-0.088953	H	-6.766732	3.875447	5.366263
C	-8.054692	-1.104469	0.302627	H	-4.503545	0.22885	5.465733
C	-6.474439	-2.860278	-0.371831	H	-3.862994	0.560514	3.108699
C	-4.182665	3.888967	1.090471	H	-2.934374	4.254932	2.808061
C	-4.960136	2.402145	2.885342	H	-2.243913	6.506557	2.07888
C	4.183296	-3.888666	1.09191	H	-4.623862	6.089577	-1.4695
C	4.960129	-2.40087	2.88627	H	-5.342161	3.852875	-0.726775
C	8.054462	1.104812	0.301991	H	8.273553	3.150091	-1.52205
C	6.47404	2.860226	-0.373083	H	7.831814	5.529969	-1.990412
C	-8.768959	-1.859473	1.244523	H	4.160863	5.286155	0.221918
C	-10.060179	-1.489083	1.612039	H	4.616744	2.916217	0.719933
C	-10.678529	-0.353296	1.072848	H	8.305256	2.736645	1.685976
C	-9.950976	0.400003	0.142131	H	10.591881	2.090652	2.346269
C	-8.666426	0.030669	-0.24952	H	10.401813	-1.287088	-0.296512
C	-7.373126	-3.618283	-1.136131	H	8.128461	-0.625118	-0.981485
C	-7.118207	-4.963049	-1.395396	H	2.934928	-4.254439	2.809527
C	-5.958469	-5.593053	-0.926263	H	2.245375	-6.506566	2.081196
C	-5.065946	-4.824778	-0.166575	H	4.625541	-6.090148	-1.467103
C	-5.317338	-3.485086	0.117607	H	5.342992	-3.852859	-0.725174
C	-5.782543	3.329072	3.542549	H	6.152917	-4.191268	3.000849
C	-6.124719	3.14411	4.879778	H	6.766642	-3.872903	5.367999
C	-5.678629	2.030342	5.603431	H	4.503398	-0.226291	5.465566
C	-4.86795	1.104798	4.933239	H	3.862931	-0.559149	3.108697
C	-4.501647	1.286771	3.601846	H	-12.31839	-0.278802	2.486647
C	-3.306693	4.656962	1.870998	H	-12.242783	1.111757	1.391906
C	-2.921647	5.929156	1.453704	H	-12.828856	-0.446803	0.805183
C	-3.372455	6.471624	0.243682	H	-6.586648	-7.605941	-1.424076
C	-4.244364	5.69349	-0.529979	H	-5.050517	-7.131502	-2.154551
C	-4.65383	4.428788	-0.116144	H	-5.117869	-7.529241	-0.43635
C	7.372715	3.618101	-1.137602	H	-7.013277	2.289329	7.290163
C	7.117712	4.962728	-1.39728	H	-6.073734	0.788316	7.334403
C	5.957865	5.592792	-0.928361	H	-5.301233	2.328688	7.718426
C	5.065406	4.824687	-0.168509	H	12.316465	0.276423	2.48836
C	5.316896	3.485062	0.1161	H	12.244013	-1.110039	1.388226
C	8.768596	1.860275	1.243648	H	12.828857	0.451532	0.808238
C	10.059802	1.490156	1.611416	H	7.011675	-2.28856	7.291815
C	10.678305	0.35419	1.072728	H	6.076725	-0.784615	7.333735
C	9.950904	-0.39954	0.14227	H	5.299327	-2.322077	7.719435
C	8.666336	-0.030478	-0.249642	H	2.638534	-8.475033	0.620911
C	3.30748	-4.656715	1.872661	H	2.041291	-7.759539	-0.879248

C	2.922961	-5.929161	1.45585	H	3.702958	-8.34686	-0.789447
C	3.374127	-6.471947	0.246028	H	-2.623351	8.469927	0.616817
C	4.245809	-5.693827	-0.527786	H	-2.048193	7.757569	-0.893537
C	4.654786	-4.428758	-0.11439	H	-3.705848	8.351982	-0.780592
C	5.782518	-3.32745	3.543963	H	6.586476	7.607675	-1.417744
C	6.124651	-3.141806	4.881126	H	5.057998	7.131188	-2.16266
C	5.678541	-2.027696	5.604203	H	5.109076	7.526558	-0.443302

Table S8. Cartesian coordinates of ICZ4B at the optimized S_0 geometry.

C	-4.273562	-4.190005	-0.284002	C	4.809378	3.320764	-0.861211
C	-2.98419	-3.65175	-0.148374	C	2.500695	2.601636	-1.270032
C	-1.763928	-4.375157	-0.137209	C	1.436864	1.665311	-1.15808
C	-1.845092	-5.762649	-0.248278	C	0.197	2.022573	-1.729769
C	-3.10914	-6.360579	-0.340728	C	-0.052162	3.250194	-2.330055
C	-4.283222	-5.602195	-0.354132	C	1.003533	4.177181	-2.353176
N	-2.70632	-2.302642	-0.018226	C	2.2539	3.868399	-1.842038
C	-1.32665	-2.10463	0.023641	C	-1.40233	3.592548	-2.914321
C	-0.714621	-0.839282	0.091765	C	5.008929	4.116781	0.268638
C	0.714568	-0.838883	-0.087906	C	5.9629	5.133258	0.245998
C	1.327424	-2.103769	-0.018728	C	6.738008	5.374543	-0.896347
C	0.698066	-3.385732	0.036999	C	6.524799	4.567064	-2.02155
C	-0.69645	-3.386227	-0.030807	C	5.572346	3.548499	-2.009047
N	2.707241	-2.300845	0.022836	C	7.789385	6.459802	-0.90503
C	2.985983	-3.649667	0.154093	B	-5.432429	-3.138594	-0.334413
C	4.27573	-4.186988	0.28961	B	5.433868	-3.134833	0.338337
C	4.286448	-5.59911	0.360817	B	-1.713911	0.303346	0.484995
C	3.112853	-6.358267	0.3486	B	1.712983	0.304088	-0.482344
C	1.848363	-5.761226	0.256239	H	-0.954728	-6.380554	-0.275824
C	1.766204	-4.37388	0.144015	H	-3.175638	-7.443085	-0.409252
C	-3.659783	-1.31205	0.056211	H	-5.227005	-6.133341	-0.408448
C	3.660087	-1.309778	-0.053381	H	5.230685	-6.129498	0.414927
C	3.196656	-0.005679	-0.323222	H	3.180079	-7.440675	0.417938
C	4.199988	0.989453	-0.483146	H	0.958422	-6.3797	0.284675
C	5.558175	0.683448	-0.291454	H	6.299205	1.459778	-0.395238
C	5.962208	-0.631619	-0.002787	H	-6.300529	1.456517	0.392184
C	5.014283	-1.689311	0.100411	H	-8.161106	0.724403	-2.085503
C	-5.013637	-1.692502	-0.098345	H	-9.811756	2.561692	-1.843216
C	-5.962048	-0.634966	0.001795	H	-10.238286	1.617571	2.3257
C	-5.558971	0.680503	0.290135	H	-8.585076	-0.21739	2.074053
C	-4.201281	0.987175	0.483843	H	-10.606382	4.319846	0.58209
C	-3.197249	-0.007427	0.32515	H	-11.794193	3.206079	1.268078
N	-7.318383	-0.92919	-0.163338	H	-11.743338	3.487741	-0.480795
N	-3.812499	2.281003	0.838532	H	-4.434328	3.932718	-1.151378

C	-8.26948	0.144949	-0.019315	H	-6.145044	5.732249	-1.11494
C	-8.616808	0.929863	-1.121437	H	-7.12303	4.706904	2.94041
C	-9.545061	1.959677	-0.977599	H	-5.410424	2.912269	2.894928
C	-10.142158	2.230148	0.261579	H	-3.049272	4.603373	1.909944
C	-9.783606	1.4317	1.355419	H	-0.854114	5.158018	2.802211
C	-8.855118	0.399408	1.221988	H	0.600286	1.289684	1.701231
C	-11.125871	3.366526	0.415827	H	-8.116587	6.739439	-0.048686
C	-4.814218	3.316308	0.869815	H	-8.65021	6.19727	1.553011
C	-2.503682	2.600444	1.272202	H	-7.351414	7.382106	1.407362
C	-5.025663	4.110796	-0.257985	H	1.893165	4.388105	2.342413
C	-5.988302	5.120065	-0.229836	H	1.313933	3.952588	3.952009
C	-6.755411	5.358006	0.917625	H	2.061016	2.723032	2.919314
C	-6.535215	4.546341	2.039507	H	-6.875825	-5.417776	-1.128512
C	-5.575345	3.535956	2.021368	H	-10.784294	-3.7048	-1.223547
C	-2.257963	3.867401	1.844274	H	-9.869175	-1.538046	-0.610512
C	-1.00799	4.1772	2.355722	H	-8.67579	-6.83033	-1.732953
C	0.048443	3.251048	2.332806	H	-9.992123	-6.011111	-2.585419
C	-0.19955	2.023352	1.732207	H	-10.181978	-6.448468	-0.887286
C	-1.439027	1.665047	1.160322	H	8.175293	0.743704	2.063279
C	-7.773576	6.473996	0.956636	H	9.832193	2.57256	1.795984
C	1.397936	3.594224	2.918129	H	10.233541	1.589479	-2.366532
C	-7.808923	-2.213496	-0.486415	H	8.574475	-0.235794	-2.090021
C	-6.941484	-3.335843	-0.602617	H	10.646004	4.256397	-0.853061
C	-7.528347	-4.563457	-0.985685	H	11.624842	3.607554	0.467169
C	-8.886418	-4.7397	-1.217998	H	11.923216	3.089683	-1.2011
C	-9.71235	-3.614287	-1.057654	H	9.870637	-1.533099	0.605709
C	-9.1949	-2.379317	-0.705881	H	10.786319	-3.697731	1.224916
C	-9.461412	-6.075285	-1.626781	H	6.877659	-5.410962	1.139855
N	3.809896	2.283144	-0.836741	H	10.183682	-6.442647	0.899168
N	7.319019	-0.92559	0.159423	H	8.678333	-6.821379	1.747746
C	8.271034	0.145831	0.002233	H	9.995533	-5.999057	2.595872
C	8.62643	0.938748	1.094929	H	-0.602188	1.288193	-1.698843
C	9.558602	1.964234	0.937086	H	0.848808	5.15783	-2.799733
C	10.15068	2.220815	-0.306405	H	3.044629	4.604965	-1.908119
C	9.782907	1.414132	-1.392292	H	-2.062483	2.719165	-2.921645
C	8.851426	0.387664	-1.245046	H	-1.318633	3.957956	-3.945726
C	11.140446	3.349189	-0.480065	H	-1.900801	4.380884	-2.333784
C	7.810198	-2.208927	0.48577	H	4.408597	3.938554	1.15599
C	9.196408	-2.374029	0.704292	H	6.105176	5.750899	1.129879
C	9.714214	-3.607822	1.059739	H	7.109356	4.739039	-2.922359
C	8.888467	-4.732659	1.224925	H	5.409888	2.930886	-2.887364
C	7.530162	-4.557234	0.993312	H	7.982735	6.823377	-1.919599
C	6.943028	-3.330963	0.606439	H	8.742996	6.092014	-0.502342
C	9.463859	-6.066748	1.638008	H	7.488916	7.315116	-0.290307

Table S9. Cartesian coordinates of ICZ4B at the optimized S_1 geometry.

C	-4.257605	-4.183337	-0.32538	C	4.841738	3.271395	-0.870773
C	-2.978844	-3.639292	-0.187745	C	2.519891	2.617148	-1.202692
C	-1.750932	-4.347855	-0.179303	C	1.447511	1.692984	-1.100543
C	-1.828971	-5.737174	-0.314631	C	0.21047	2.090065	-1.633982
C	-3.086693	-6.336059	-0.416401	C	-0.029184	3.344205	-2.175724
C	-4.267107	-5.595213	-0.417214	C	1.036823	4.258283	-2.186536
N	-2.701339	-2.288774	-0.044175	C	2.287686	3.909232	-1.718849
C	-1.327154	-2.091517	0.002537	C	-1.382323	3.731848	-2.698359
C	-0.720551	-0.821267	0.087576	C	5.107808	4.043646	0.257225
C	0.720586	-0.821254	-0.087083	C	6.099485	5.016449	0.211143
C	1.327224	-2.091489	-0.002042	C	6.84037	5.238729	-0.953709
C	0.703812	-3.368848	0.051353	C	6.561603	4.451508	-2.073956
C	-0.703705	-3.368869	-0.050797	C	5.570915	3.475893	-2.039204
N	2.701428	-2.28872	0.04457	C	7.890089	6.313296	-1.003018
C	2.978969	-3.639232	0.188185	B	-5.411565	-3.140517	-0.360115
C	4.257749	-4.183252	0.325734	B	5.411695	-3.140418	0.360279
C	4.267285	-5.595124	0.417645	B	-1.702002	0.301356	0.470892
C	3.08688	-6.335981	0.416969	B	1.701981	0.301372	-0.470494
C	1.829139	-5.73712	0.315261	H	-0.935187	-6.348808	-0.356253
C	1.751066	-4.347809	0.179868	H	-3.144013	-7.417861	-0.503765
C	-3.648822	-1.309922	0.049947	H	-5.207668	-6.130987	-0.474807
C	3.648876	-1.30987	-0.049712	H	5.207861	-6.130879	0.475177
C	3.191731	-0.012868	-0.328357	H	3.144218	-7.417779	0.504381
C	4.189737	0.971594	-0.478375	H	0.935368	-6.348765	0.35697
C	5.549032	0.663171	-0.290091	H	6.29349	1.439914	-0.387273
C	5.947947	-0.65403	0.001252	H	-6.293507	1.439866	0.386988
C	5.000437	-1.694959	0.109253	H	-8.131207	0.71564	-2.072329
C	-5.000352	-1.695033	-0.10916	H	-9.75226	2.576555	-1.806407
C	-5.947886	-0.654086	-0.001387	H	-10.1582	1.616473	2.353323
C	-5.549027	0.663126	0.28995	H	-8.534155	-0.241153	2.077469
C	-4.189769	0.971561	0.478437	H	-10.484889	4.313487	0.675999
C	-3.191726	-0.012903	0.328578	H	-11.714321	3.208376	1.294611
N	-7.296132	-0.938099	-0.165303	H	-11.615599	3.547142	-0.440358
N	-3.82246	2.265798	0.819487	H	-4.53551	3.872116	-1.163398
C	-8.235018	0.133953	-0.011435	H	-6.303771	5.613502	-1.095838
C	-8.577971	0.925062	-1.105137	H	-7.129196	4.602527	2.989325
C	-9.486761	1.965083	-0.947654	H	-5.357476	2.865336	2.911949
C	-10.066854	2.23635	0.295116	H	-3.094784	4.630274	1.772074
C	-9.713695	1.428269	1.379252	H	-0.884801	5.258275	2.587163
C	-8.804763	0.385933	1.233405	H	0.600097	1.367506	1.607747
C	-11.0241	3.382234	0.464641	H	-8.450829	6.368622	0.066546

C	-4.841838	3.271308	0.871086	H	-8.600374	6.141816	1.817891
C	-2.520003	2.617111	1.202999	H	-7.437297	7.298321	1.168473
C	-5.108053	4.043715	-0.256732	H	1.869236	4.451131	2.029574
C	-6.099777	5.016506	-0.210376	H	1.314334	4.202426	3.686425
C	-6.840496	5.238596	0.954575	H	2.035587	2.859344	2.783713
C	-6.56162	4.451143	2.074675	H	-6.852977	-5.396142	-1.174975
C	-5.570934	3.475586	2.039645	H	-10.759431	-3.681001	-1.232038
C	-2.287876	3.909175	1.719233	H	-9.839212	-1.519576	-0.596808
C	-1.037091	4.258211	2.18715	H	-8.660223	-6.789987	-1.783385
C	0.028908	3.344129	2.176508	H	-9.970105	-5.95255	-2.625803
C	-0.210659	2.090016	1.634668	H	-10.16254	-6.406497	-0.933843
C	-1.447609	1.69296	1.10099	H	8.131884	0.716424	2.071161
C	-7.88999	6.313352	1.004503	H	9.752755	2.577345	1.804015
C	1.381939	3.731723	2.699467	H	10.157452	1.615691	-2.355474
C	-7.78519	-2.206934	-0.495045	H	8.533595	-0.241893	-2.07841
C	-6.919539	-3.325681	-0.626122	H	10.484637	4.312131	-0.685813
C	-7.506939	-4.544716	-1.021011	H	11.611196	3.551706	0.438844
C	-8.862485	-4.712784	-1.248845	H	11.717614	3.205376	-1.294182
C	-9.687428	-3.590357	-1.070539	H	9.839373	-1.519413	0.596138
C	-9.170353	-2.364405	-0.707672	H	10.759685	-3.680753	1.231539
C	-9.440789	-6.033369	-1.669589	H	6.853219	-5.395907	1.17529
N	3.822397	2.265829	-0.819374	H	10.162752	-6.406284	0.933823
N	7.296223	-0.938008	0.16494	H	8.660566	-6.789656	1.78365
C	8.235077	0.133988	0.010462	H	9.970572	-5.952083	2.625743
C	8.578329	0.925496	1.103746	H	-0.600294	1.36757	-1.606913
C	9.487023	1.965536	0.945578	H	0.884459	5.258362	-2.586486
C	10.066689	2.236376	-0.297453	H	3.094588	4.630329	-1.771802
C	9.713255	1.427835	-1.381193	H	-2.035925	2.859451	-2.782755
C	8.804441	0.385529	-1.23467	H	-1.314905	4.202861	-3.68518
C	11.023714	3.382304	-0.467919	H	-1.869562	4.451012	-2.028162
C	7.785338	-2.206794	0.494768	H	4.535137	3.871899	1.163782
C	9.170535	-2.364231	0.707211	H	6.303343	5.613323	1.096722
C	9.68766	-3.590129	1.070178	H	7.12923	4.6031	-2.988536
C	8.862739	-4.712533	1.248769	H	5.357546	2.865823	-2.911656
C	7.50716	-4.544505	1.021108	H	8.59584	6.146349	-1.821357
C	6.919702	-3.325528	0.626124	H	8.456224	6.362542	-0.067879
C	9.44111	-6.033052	1.669623	H	7.437006	7.299477	-1.158308
